# Deep learning model and omics screening highlight angiotensinogen as a 5-methylcytosine (m^5^C) regulated mediator of tumor-microenvironment communication in liver cancer

**DOI:** 10.3389/fimmu.2026.1752802

**Published:** 2026-02-20

**Authors:** Huai Pang, Linshu Wang, Yang Li, Yuan Zhou

**Affiliations:** 1 Department of Biomedical Informatics, School of Basic Medical Sciences, Peking University, Beijing, China; 2 Department of Cell Biology, School of Basic Medical Sciences, Peking University Stem Cell Research Center, Peking University, Beijing, China; 3 State Key Laboratory of Vascular Homeostasis and Remodeling, Peking University, Beijing, China

**Keywords:** angiotensinogen, deep learning, liver cancer, m5C modification, natural killer cell, tumor microenvironment

## Abstract

**Introduction:**

The tumor microenvironment (TME) is critical for liver cancer progression and therapy response. As a key RNA modification, 5-methylcytosine (m^5^C) methylation is implicated in this process, yet the molecular mechanisms by which m^5^C modification mediates intercellular crosstalk within the TME remain less understood.

**Methods:**

The m^5^C methylomes in wildtype and m^5^C-catalyzing enzyme *NSUN2*-perturbed liver cancer cells were profiled via MeRIP-seq. GAT-MeRIP, a graph attention neural network-based algorithm, was developed to identify functional m^5^C-modified target genes from MeRIP-seq data. TME-related functional m^5^C targets were screened through cell-cell communication analysis of single-cell transcriptomic data. *In vitro* functional validation of the key target gene was performed via a combination of cell co-culture, qRT-PCR, MeRIP-qPCR, ELISA, and flow cytometry assays. Additionally, public liver cancer cohort data were used for clinical correlation and prognostic analysis.

**Results:**

Angiotensinogen (AGT) was identified as a key m^5^C-regulated secretory factor contributing to tumor-microenvironment communication in liver cancer. NSUN2 knock-down increased AGT’s expression and enhanced cytotoxicity of co-cultured NK cells, which can be canceled by AGT-neutralizing antibody. Exogenous AGT treatment significantly enhanced NK cell cytotoxicity by upregulating IFN-γ, TNF-α, and perforin, as well as the proportion of CD107a⁺ NK cells. Liver cancer patients with low NSUN2 and high AGT exhibited significantly improved overall survival rates and higher immune infiltration.

**Conclusions:**

This study unveils novel regulatory function of m^5^C-modified AGT in modulating the liver TME that could be helpful for improving liver cancer prognosis and immunotherapy.

## Introduction

Liver cancer refers to malignant tumors occurring in the liver, ranking sixth in incidence and third in mortality among all cancers worldwide (1–3). Early-stage liver cancer often develops insidiously without obvious symptoms, and clinical manifestations in the middle and late stages frequently lack specificity (4). Despite significant advances in early prevention, diagnostic, and precision medicine, the five-year overall survival rate remains suboptimal due to the biological characteristics of liver cancer, including its tendency to recur, metastasize distantly, and develop drug resistance (5–7). The tumor microenvironment (TME) is a structured ecosystem composed of diverse immune cells, cancer-associated fibroblasts, endothelial cells, and numerous other tissue-resident cells, involving complex intercellular communication (8, 9). The TME in liver cancer exhibits high heterogeneity, significantly impacting the efficacy of radiotherapy, chemotherapy, and immunotherapy in cancer patients, leading to tumor recurrence and the development of drug resistance (9–11). Therefore, insights in the molecular mechanisms of communication between cells within the TME of liver cancer will contribute to the prevention, diagnosis, and treatment of this disease.

5-methylcytosine (m^5^C) methylation is a widespread modification found in various RNA molecules that can influence RNA stability, transcription, and translation processes (12–14). NSUN2 is a member of the NOP2/Sun-domain (NSUN) family and an important m^5^C methyltransferase for modifications on mRNAs (14, 15). Multiple experimental findings indicate that NSUN2 is highly expressed in liver cancer and promotes the proliferation, invasion and migration of liver cancer by targeting different pathways in tumor cells (16–18). However, the molecular role of NSUN2 in the intercellular communication between tumor cells and TME remains largely unknown. Currently, through high-throughput RNA m^5^C methylome profiling technique such as RNA m^5^C methylation immunoprecipitation sequencing (m^5^C-MeRIP-seq) and bisulfite sequencing, the m^5^C target genes can be experimentally identified at the whole transcriptome scale. Moreover, with the advancement of deep learning, various neural network-based m^5^C prediction algorithms have emerged, such as the Trans-m^5^C model (19), m^5^C-iDeep model (20), and Deepm^5^C model (21). Compared to traditional machine learning-based prediction methods, these algorithms have demonstrated significantly improved predictive performance for *in silico* identification of m^5^C-modified target genes. However, neither experimental nor computational approaches above could not directly prioritize the key functional m^5^C targets among thousands of candidate target genes.

**Figure 1:**
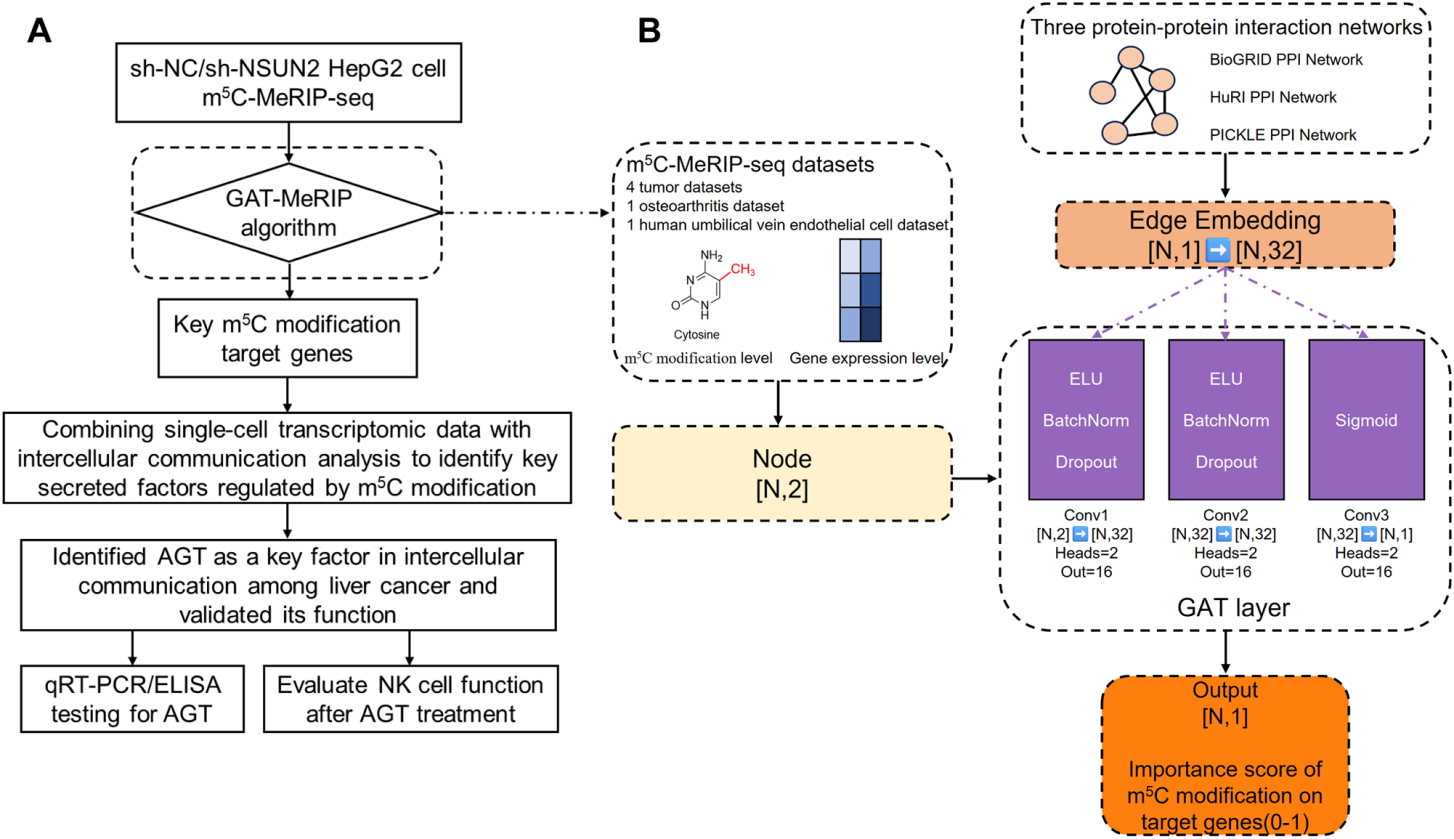
Flowchart of the study and GAT-MeRIP algorithm framework. **(A)** Flowchart summarizing this study. **(B)** Flowchart depicting the algorithm framework of GAT-MeRIP.

To this end, we here developed GAT-MeRIP, a method for screening key m^5^C-methylated target genes based on graph attention neural network (GAT) deep learning framework. Subsequently, we profiled m^5^C-MeRIP-seq data from NSUN2 knock-down (sh-NSUN2) and control (sh-Control) liver cancer cells, and identified potentially key functional m^5^C target genes using the GAT-MeRIP algorithm. By further integrating single-cell transcriptomic data from liver cancer and intercellular communication analysis, we identified angiotensinogen (AGT) as a key m^5^C target that participates in cell-cell communication, and experimentally validated the role of AGT in mediating TME by various *in-vitro* assays (**Figure 1A**).

## Materials and methods

### GAT-MeRIP algorithm framework

The computational framework of GAT-MeRIP is illustrated in **Figure 1B**. We collected RNA m^5^C modification data of multiple cell lines and disease contexts from the gene expression omnibus (GEO, https://www.ncbi.nlm.nih.gov/geo/), including GSE221793, GSE267662, GSE280936, GSE246721, GSE249045, and GSE235610. Each dataset comprised paired control and treatment groups subjected to RNA m^5^C immunoprecipitation sequencing (m^5^C-MeRIP-seq). Raw reads were processed by fastp software (version 0.20.1) and aligned to the human reference genome (UCSC hg38) using HISAT2 (version 2.2.1) with default parameters. Gene annotations were obtained from UCSC refGene database (version 2020.01). The resulting BAM files were directly processed by exomePeak2 R package (version 1.16.2) (22) for subsequent peak calling and quantification. The exomePeak2 internally performs read counting and normalization, converting raw read counts to reads per million (RPM)-normalized gene expression levels. We performed peak calling separately on the control and treatment groups for each sample, and the workflow generated two key metrics for each gene: gene expression level and m^5^C modification level. For genes with multiple m^5^C peaks, we aggregated duplicate gene entries and filtered low-expression genes (< 1.0 RPM) or weak m^5^C enrichment (< 0.1 RPM). Then gene expression and m^5^C modification levels were further scaled using Z-score standardization within each dataset and served as the input of the model.

Graph attention network (GAT) is a type of graph neural network that could effectively exploits the functional relationship between genes via custom gene graph (23, 24). Here, a comprehensive protein-protein interaction (PPI) network was employed as the gene graph constraint in GAT model. To construct a high-quality PPI network, we integrated interaction data from three databases: BioGRID (https://thebiogrid.org), HuRI (http://www.interactome-atlas.org), and PICKLE (http://www.pickle.gr). During network construction, each PPI was represented by two directed edges to support message propagation while preserving the underlying undirected biology, each assigned an equal weight (weight = 1). The core of the neural network framework is a multi-layer GAT architecture comprising three cascaded graph attention layers, each meticulously designed to capture gene regulatory relationships at distinct levels. The first GAT layer features two attention heads with an input dimension of 2 (corresponding to gene expression and m^5^C modification levels) and an output dimension of 16. Through the multi-head attention mechanism, each attention head independently learns feature representations of different aspects. This parallel feature extraction approach significantly enhances the model’s ability to capture complex patterns. The second GAT layer also employs two attention heads, but the input dimension increases to 32 (derived from the 16-dimensional output of the first layer multiplied by two heads), while maintaining a 16-dimensional output. This design enables the model to learn gene interactions within a higher-dimensional feature space. The third layer, serving as the output layer, employs a single attention head to compress features into a 1-dimensional output, yielding importance scores for each gene. To enhance expressive power and training stability, several key components are introduced into the network. First, a 32-dimensional edge embedding layer precedes each GAT layer, converting raw edge type information into dense vector representations. This embedding approach enables the model to better leverage structural information within protein interaction networks. Second, batch normalization layers are added after the first and second GAT layers, accelerating model convergence while providing regularization. The exponential linear unit (ELU) activation function was chosen over traditional rectified linear unit. ELU mitigates the issue of neuron death and provides non-zero gradients in the negative range. Additionally, a dropout rate of 0.3 was applied after each layer to prevent overfitting while preserving the model’s expressive power. For model training, we merged data from all six datasets and split them into training (80%) and validation (20%) sets using stratified random sampling to maintain dataset distribution. Adam optimizer was used with an initial learning rate of 5e-4 and weight decay of 1e-4, demonstrating excellent convergence performance. The loss function utilizes binary cross-entropy, as the task is fundamentally a binary classification problem: determining whether a gene is a key regulator of m^5^C modification. Genes with detected m^5^C peaks were labeled as positive samples (y = 1), while all other genes in the PPI network without detected modification were labeled as negative samples (y = 0). This classification labelling strategy prevents leakage of knowledge about known key functional genes, which was used as the ground-truth labels in model evaluations. To avoid overfitting, an early stopping mechanism based on validation set loss is implemented. Training automatically terminates when the validation loss fails to improve for 30 consecutive epochs. This mechanism effectively balances model fit and generalization capability. During each training epoch, the average loss on both the training and validation sets is computed. These metrics not only inform early stopping decisions but also facilitate monitoring the model’s learning process. The model was trained for a maximum of 200 epochs, with the best-performing model (lowest validation loss) saved.

### Cell lines and cell culture

Human liver cancer cell lines HepG2 and JHH-7 were obtained from Wuhan Procell Biotechnology Co., Ltd. (Wuhan, China), and cultured separately in Dulbecco’s Modified Eagle Medium (DMEM; STEEMA, China) and DMEM/F12 (STEEMA, China) supplemented with 10% fetal bovine serum (Vivacell, China) and 1% penicillin-streptomycin solution (Gibco, USA). Human NK cell line YT (YT-NK) was maintained under the same culture conditions. Peripheral blood mononuclear cells (PBMCs) were purchased from Milestone^®^ Biotechnologies (Shanghai, China), and NK cells were expanded *in vitro* for two weeks using the Natural Killer Cell Induction Culture Kit 2.0 (Dakewe, China). The expanded NK cells were characterized via flow cytometry. All cell lines were incubated in a humidified atmosphere at 37 °C with 5% CO_2_. To ensure experimental reproducibility, cell line authentication was performed via short tandem repeat (STR) profiling, and mycoplasma contamination testing was routinely conducted. Experiments were carried out exclusively with mycoplasma-free cultures.

### NSUN2 knock-down through lentivirus infection

Lentiviral vectors targeting NSUN2 were purchased from Syngentech Co., Ltd. (Beijing, China). The NSUN2 knock-down constructs were designated as sh-NSUN2 (#1: 5’-GAAACACCGAGCGATGCCTTA-3’; #2: 5’-GCTTGGACTACCATATGAA CT-3’), while a non-targeting control vector was labeled sh-NC. Cells were transduced with lentiviral particles following the manufacturer’s protocol. At 48 hours post-transduction, the culture medium was replaced with fresh medium supplemented with a high concentration of puromycin for selective screening. NSUN2 knock-down efficiency was subsequently confirmed by qRT-PCR and western blot analysis (**Supplementary Figures 1A, B**).

### RNA m^5^C methylation immunoprecipitation sequencing

Total RNA from sh-NC and sh-NSUN2 HepG2 cells was fragmented and enriched for m^5^C modifications using anti-m^5^C antibody (Diagenode, Belgium) via immunoprecipitation. Enriched RNA fragments were converted to cDNA libraries and sequenced as paired-end 150 bp reads at Aksomics Inc. (Shanghai, China). Raw reads were processed by fastp (version 0.20.1), and aligned to the human reference genome (UCSC hg38) using HISAT2 (version 2.2.1) with default parameters. Gene annotations were obtained from UCSC refGene database (version 2020.01). The resulting BAM files were directly processed by exomePeak2 R package (version 1.16.2) for subsequent peak calling and quantification. Differential m^5^C peaks were identified using the exomePeak2 R package with the criteria of 1.5-fold change and p-value < 0.05. Peak distribution and gene association were visualized using the GenomicRanges (version 1.56.2) and ggplot2 (version 3.5.2) R packages. The m^5^C motifs were identified by using the STREME method (https://meme-suite.org/meme/tools/streme).

### RNA extraction and quantitative real-time polymerase chain reaction

Total RNA was isolated using TRIzol^®^ reagent (Thermo Fisher Scientific, Waltham, USA) following the manufacturer’s guidelines. For cultured cells, 1 mL of TRIzol per 10^6^ cells was used to lyse the cells, followed by a 30-minute incubation at 4°C. Chloroform (0.2 mL per 1 mL TRIzol) was added to facilitate phase separation, and the mixture was centrifuged at 12, 000 × g for 15 minutes at 4°C. The upper aqueous layer containing RNA was transferred to a new tube and precipitated by adding isopropanol (0.5 mL per 1 mL TRIzol), then incubated at 4°C for 10 minutes. After centrifugation at 12, 000 × g for 10 minutes at 4°C, the RNA pellet was washed twice with 75% ethanol, air-dried, and resuspended in RNase-free water for downstream applications. RNA purity and concentration were measured using a NanoDrop™ 2000 spectrophotometer, with integrity confirmed by 1% agarose gel electrophoresis. For cDNA synthesis, 1 μg total RNA was reverse-transcribed using the Revert Aid First-Strand cDNA kit (Thermo Fisher Scientific, USA) according to the manufacturer’s protocols. qRT-PCR was performed in triplicate on the Rotor-Gene Q MDx detection system (QIAGEN, Germany) using the SYBR Green PCR kit (QIAGEN, Germany) to detect mRNA. Data were analyzed using the 2^−ΔΔCq^ method normalized to β-Actin (primers listed in **Supplementary Table 1**).

### Methylated RNA immunoprecipitation qRT-PCR assay

m^5^C-modified RNA was enriched using the GenSeq^®^ m^5^C MeRIP Kit (CloudSeq, China) following the manufacturer’s instructions. Total RNA was isolated and fragmented (~100 nt) from HepG2 cells, followed by immunoprecipitation with anti-m^5^C antibody (4 °C overnight). RNA-antibody complexes were captured by Protein A/G magnetic beads, washed, and eluted. Both input and immunoprecipitated RNA fractions were reverse-transcribed into cDNA, and m^5^C-enriched AGT were quantified by qRT-PCR.

### RNA stability analysis

HepG2 cells were treated with the transcription inhibitor actinomycin D (5µg/mL; Beyotime Biotechnology, China) and harvested at 0, 3, and 6hours post-treatment. Total RNA was extracted from each time point, and AGT mRNA levels were quantified by qRT-PCR. RNA stability was assessed by calculating the percentage of remaining AGT mRNA at each time point relative to its level at 0hours.

### Cell co-culture and NK cytotoxicity tests (LDH release-based)

AGT recombinant protein (100 ng/mL) was used to pretreat NK cells for 24 hours at 37 °C (5% CO_2_) prior to co-culture. Target cells (5×10³/well) were seeded in 96-well plates and cultured overnight, then co-cultured with AGT-treated NK cells at various effector-to-target ratios for 4 hours under identical conditions. Supernatants were centrifuged (300g, 5 min) to remove debris, and LDH release was measured using the LDH Cytotoxicity Assay Kit (Beyotime, China) following manufacturer instructions. Cytotoxicity was calculated as​: %Cytotoxicity = [(Experimental − Effector Spontaneous − Target Spontaneous)/(Target Maximum − Target Spontaneous)] × 100.

### Enzyme linked immunosorbent assay

Cell culture supernatants were collected 72 hours post-treatment, centrifuged at 300g for 10 min to remove debris, aliquoted, and stored at -80 °C. IFN-γ and TNF-α were measured using High Sensitivity ELISA Kit (MULTI SCIENCES, China) following the manufacturer’s protocol for antibody incubation, colorimetric development, and optical density measurement. Secretion levels were quantified using standard curves.

### Flow cytometric analysis

To assess CD107a expression on NK cells, CD107a surface expression was tested using a PE-conjugated anti-CD107a antibody (1:200; BioLegend, USA). Briefly, single-cell suspensions were stained with fluorophore-labeled antibodies for 30 minutes at 4°C, washed, and analyzed via flow cytometry (BD FACSymphony™ S6, USA). Live NK cells were gated based on forward/side scatter (FSC/SSC), and the proportion of CD107a^+^ NK cells was quantified using FlowJo™ software.

### Processing and re-analysis of liver cancer single-cell RNA sequencing data

The scRNA-seq data were obtained from Mendeley Data (https://doi.org/10.17632/skrx2fz79n.1). All analyses were performed using R version 4.4.0. The Seurat object was constructed and analyzed using Seurat (version 5.3.0). Gene expression data were normalized using the LogNormalize method with a scale factor of 10, 000. Highly variable features (n=2, 000) were identified using the variance-stabilizing transformation (VST) method. Data were then scaled using the *ScaleData* function across all genes. Principal component analysis (PCA) was performed on the scaled data using the top 2, 000 variable features. The optimal number of principal components (PCs) was determined by examining the elbow plot, and 30 PCs were selected for downstream analysis. Uniform Manifold Approximation and Projection (UMAP) dimensionality reduction was performed with default parameters. Cell clustering was performed using the Louvain algorithm with multiple resolutions (0.3, 0.5, 0.8, 1.0, and 1.2) tested to identify optimal clustering granularity. A resolution of 0.8 was selected for detailed analysis, yielding 40 distinct clusters. Differentially expressed genes (DEGs) for each cluster were identified using the *FindAllMarkers* function. Then cell type identities were validated by examining the expression of canonical marker genes. Cell-cell communications between tumor cells and immune cells were analyzed using the CellChat (version 1.6.1) and nichenetr (version 2.2.0) R packages.

### Collection and analysis of public liver cancer patient datasets

RNA-seq and clinicopathological data of liver cancer were obtained from the GEO database (https://www.ncbi.nlm.nih.gov/geo/) and The Cancer Genome Atlas (TCGA) database (https://portal.gdc.cancer.gov/). GSE214846 was employed for AGT-NSUN2 correlation analysis. The immunotherapy data from GSE272035, GSE235863 and GSE279750 were utilized to compare AGT expression before and after treatment. The liver cancer cohort from TCGA underwent survival analysis using R packages Survival (version 3.5.8), along with immune infiltration analysis via R packages GSVA (version 2.0.1), xCell (version 1.1.0), Tumor Immune Dysfunction and Exclusion (TIDE, http://tide.dfci.harvard.edu/). AGT expression in liver cancer progression was assessed using the online tool TNMplot (https://tnmplot.com/analysis/).

### Statistical analysis

All statistical analyses were performed using the R software (version 4.3.1), GraphPad Prism 8.0, or SPSS 20.0 software. Data are presented as mean ± standard deviation (SD) of at least three independent experiments. Student’s t-test was used to compare the mean values of normally distributed data between groups, and Mann-Whitney U-test was used for non-normally distributed data. A *P*-value less than 0.05 was considered statistically significant.

## Results

### GAT-MeRIP prioritized functional genes in a more robust and efficient manner

**Figure 2:**
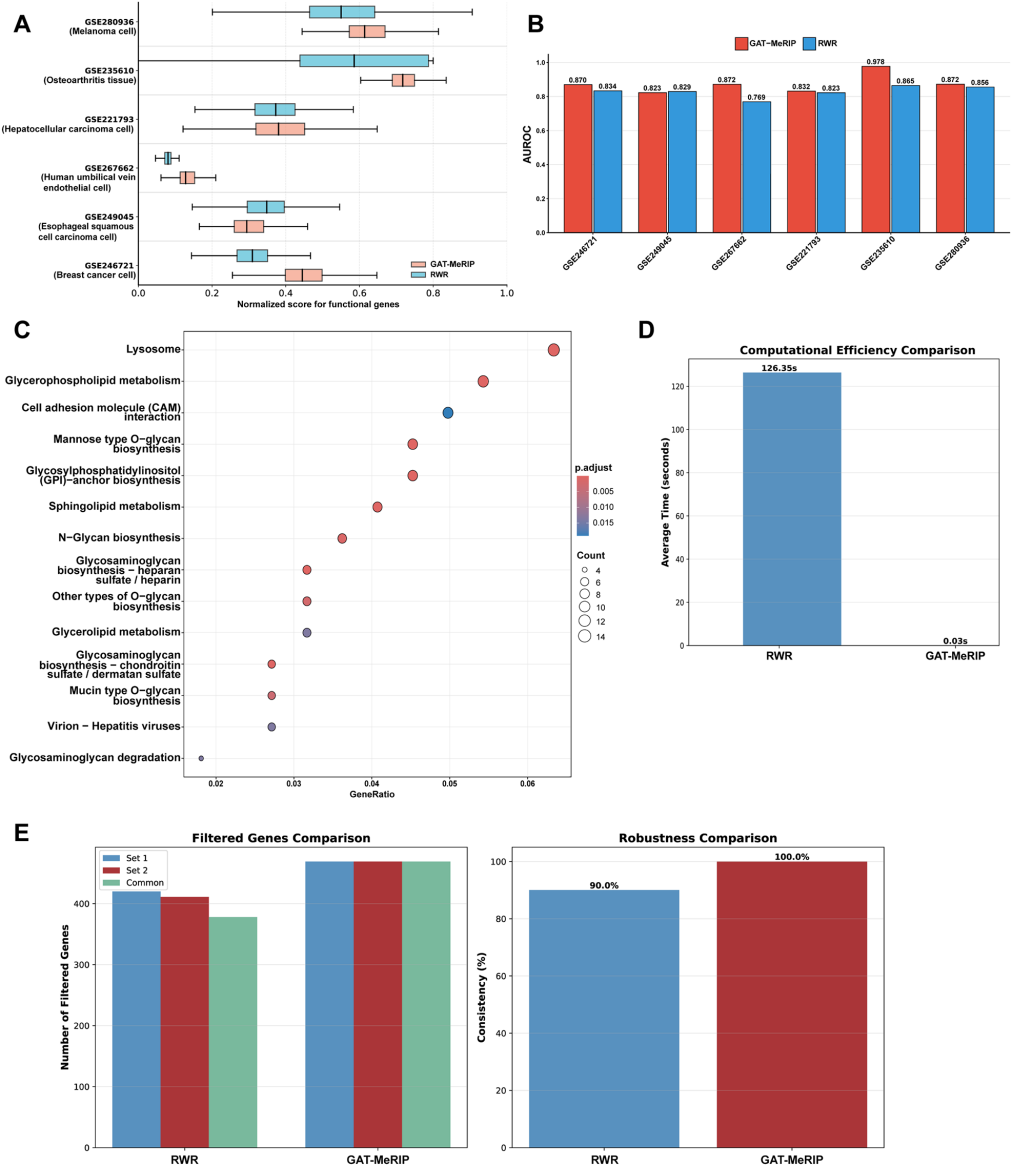
Comparison of GAT-MeRIP and RWR. **(A)** To ensure comparability, gene scores from both GAT-MeRIP and RWR algorithms are normalized to the [0, 1] range using Min-Max and Z-score normalization, and their functional gene scores are compared. **(B)** Comparison of AUROC values of algorithms in functional gene identification tasks. **(C)** KEGG enrichment analysis is performed on the top 200 genes ranked by GAT-MeRIP scores across four cancer datasets. **(D)** Comparison of algorithm effectiveness. **(E)** Comparison of algorithm robustness.

To evaluate the algorithm’s ability in prioritizing known functional m^5^C target genes, we compared GAT-MeRIP with random walk with restart (RWR), which is the approach used in m^6^A-Driver (25) to identify key target gene of RNA modification. We compared two methods on six independent m^5^C-MeRIP-seq datasets. GAT-MeRIP scored m^5^C-modified genes based on their network context and modification patterns, while RWR propagated scores from 500 randomly selected m^5^C-modified genes across the PPI network. To ensure comparability, the gene scores were all normalized to [0, 1] via Min-Max normalization and Z-score normalization. Cancer driver genes collected from COSMIC (https://cancer.sanger.ac.uk/cosmic/), IntOGen (https://www.intogen.org/) and disease gene collected from DisGeNET (https://disgenet.com/) were introduced as the ground-truth. The results indicated that GAT-MeRIP was more effective than RWR in both functional gene scoring and AUROC values, suggesting successful prioritization of key functional m^5^C targets (**Figures 2A, B**). We further performed KEGG enrichment analysis of genes with high GAT-MeRIP scores (top 200) across four cancer-related m^5^C-MeRIP-seq datasets (GSE246721, GSE249045, GSE221793, and GSE280936). The results revealed significant enrichment in pathways related to glycosylation, lipid metabolism, and cell adhesion molecule function (**Figure 2C**). These pathways are all closely associated with tumorigenesis and progression. Together, all results collectively demonstrated that the GAT-MeRIP efficiently identifies functionally important genes.

To further evaluate the robustness and efficiency of GAT-MeRIP in filtering key functional genes, we compared GAT-MeRIP with RWR (**Figures 2D, E**). Similar to the strategy used in m^6^A-Driver, we randomly selected 100 m^5^C-modified genes from our m^5^C-MeRIP-seq data as seeds and applied both GAT-MeRIP and RWR on two different sets of random networks (100 networks per set) that preserve the topological properties of the PPI network. As shown in **Figures 2E**, GAT-MeRIP demonstrated superior robustness, with 0 different candidate genes filtered between the two random network sets, compared to 75 genes for RWR. This indicates that GAT-MeRIP produces more consistent results across different network configurations. Moreover, GAT-MeRIP achieved a 4212-fold speedup over RWR (**Figure 2D**), as it only requires the degree information of candidate and seed genes rather than performing iterative random walks on each network.

### Screening key m^5^C target gene involved in tumor-microenvironment communication of liver cancer

**Figure 3:**
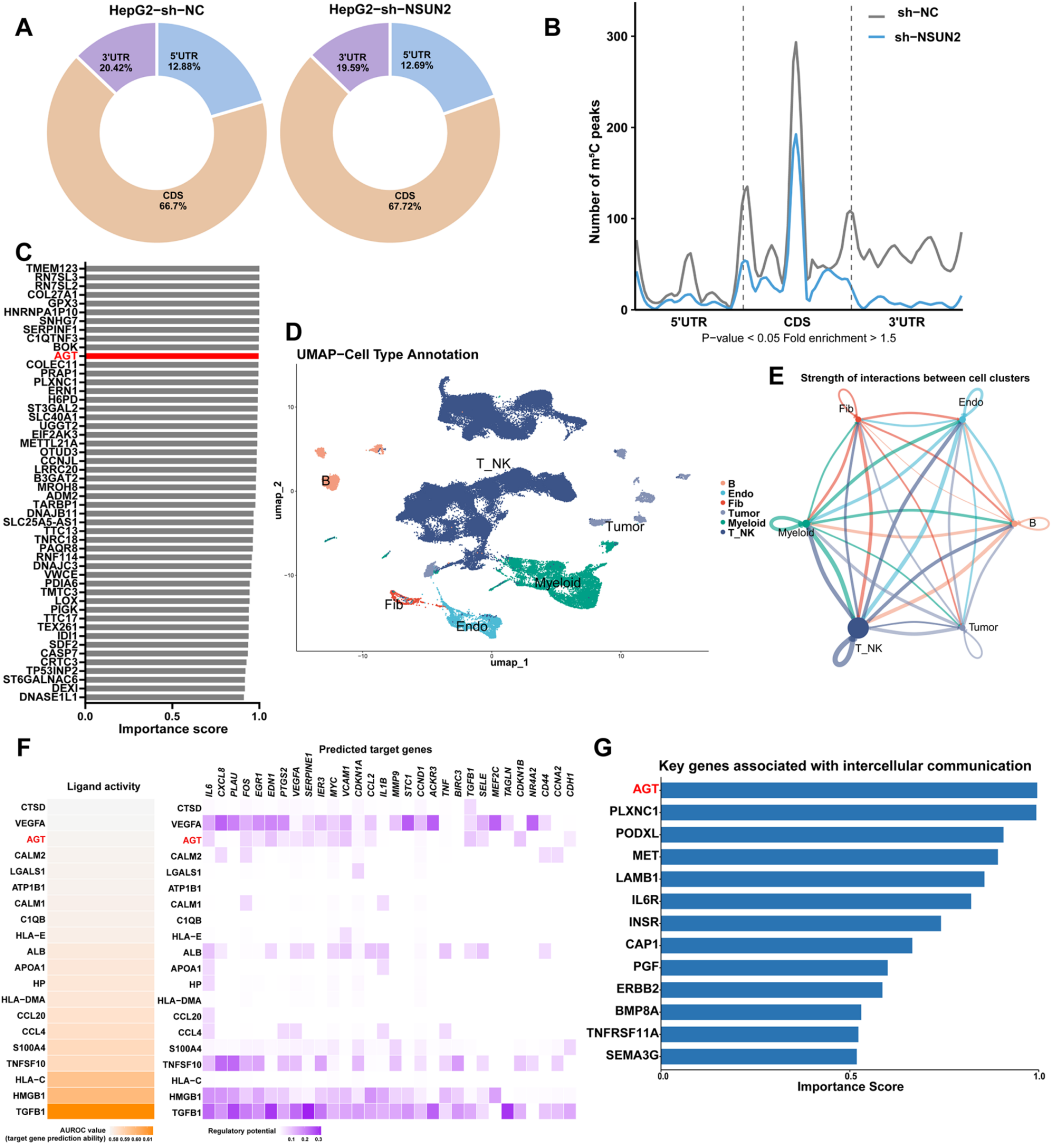
Identify AGT as a key factor in intercellular communication among liver cancer. **(A)** Distribution of m^5^C peaks in different regions of mRNA as detected in m^5^C-MeRIP-seq assays conducted in sh-NC or sh-NSUN2 HepG2 cells. **(B)** The significantly decreased (blue) m^5^C peaks in sh-NSUN2 HepG2 cells. **(C)** The key m^5^C-modified target genes and their scores obtained through GAT-MeRIP model. Top 50 genes are shown. **(D)** UMAP plot showing the cell type annotations. **(E)** Analyze the strength of interactions between cell groups using CellChat. **(F)** Heatmaps visualization of NicheNet-inferred ligand activity of top-ranking ligands expressed by liver cancer cells (left) and their regulatory potential on predicted target genes expressed by immune cells (right). **(G)** Key m^5^C-modified target genes associated with cellular communication and their scores. B, B cells; Endo, endothelial cells; Fib, fibroblasts; Tumor, liver cancer cells; Myeloid, myeloid cells; T_NK, T cells and NK cells.

To screen key m^5^C target gene in liver cancer, we firstly profiled the m^5^C peaks in sh-NC or sh-NSUN2 HepG2 cells through m^5^C-MeRIP-seq technique. The vast majority of m^5^C peaks were distributed in the coding sequence (CDS) and 3’untranslated regions (UTR) (**Figure 3A**). The consensus motifs of m^5^C peaks could be identified in the peak region (**Supplementary Figure 2A**). NSUN2 knock-down induced an obvious decrease of m^5^C peaks (**Figure 3B**). More specifically, there were 1477 upregulated peaks and 545 downregulated peaks in sh-NC compared to sh-NSUN2 in HepG2 cells (**Supplementary Figure 2B**). KEGG enrichment analysis of m^5^C-modified differentially expressed genes revealed enrichment in pathways related to protein hydrolysis and endoplasmic reticulum biogenesis (**Supplementary Figure 2C**). Subsequently, a set of key m^5^C-modified target genes was identified from the m^5^C-MeRIP-seq data using above-mentioned GAT-MeRIP algorithm (**Figure 3C**; **Supplementary Table 2**).

To further identify intercellular communication factors between tumor cells and TME, scRNA-seq data from liver cancer was analyzed. We identified and visualized 40 clusters using the UMAP method, annotating six distinct cell types (**Figure 3D**, **Supplementary Figure 3A**). We further performed intercellular communication analysis on the scRNA-seq data, the results indicate extensive interactions between tumor cells and different TME cell groups (**Figure 3E**). When focusing on the interactions between tumor cells and T_NK cells, top ligand proteins from tumor cells that are likely to mediate tumor-T_NK communications were identified, where AGT was the only protein show top-ranked cell-cell communication signature among the important m^5^C targets selected by GAT-MeRIP (**Figure 3F**). Concurrently, among the proteins mediating intercellular communication, AGT ranked the top in the m^5^C target importance score estimated by GAT-MeRIP (**Figure 3G**). UMAP visualization also confirm that, while several tumor cell clusters could be identified due to patient heterogenicity, AGT showed enriched expression across nearly all of the tumor cell clusters (**Supplementary Figure 3B–D**). Together, the results prioritized AGT as a key m^5^C-modified secretory factor primarily expressed in liver cancer cells, potentially playing a crucial role in cell-cell communication within liver cancer, which is experimentally validated in the next sections.

### AGT expression was negatively correlated with NSUN2 and positively correlated with NK cell infiltration

**Figure 4:**
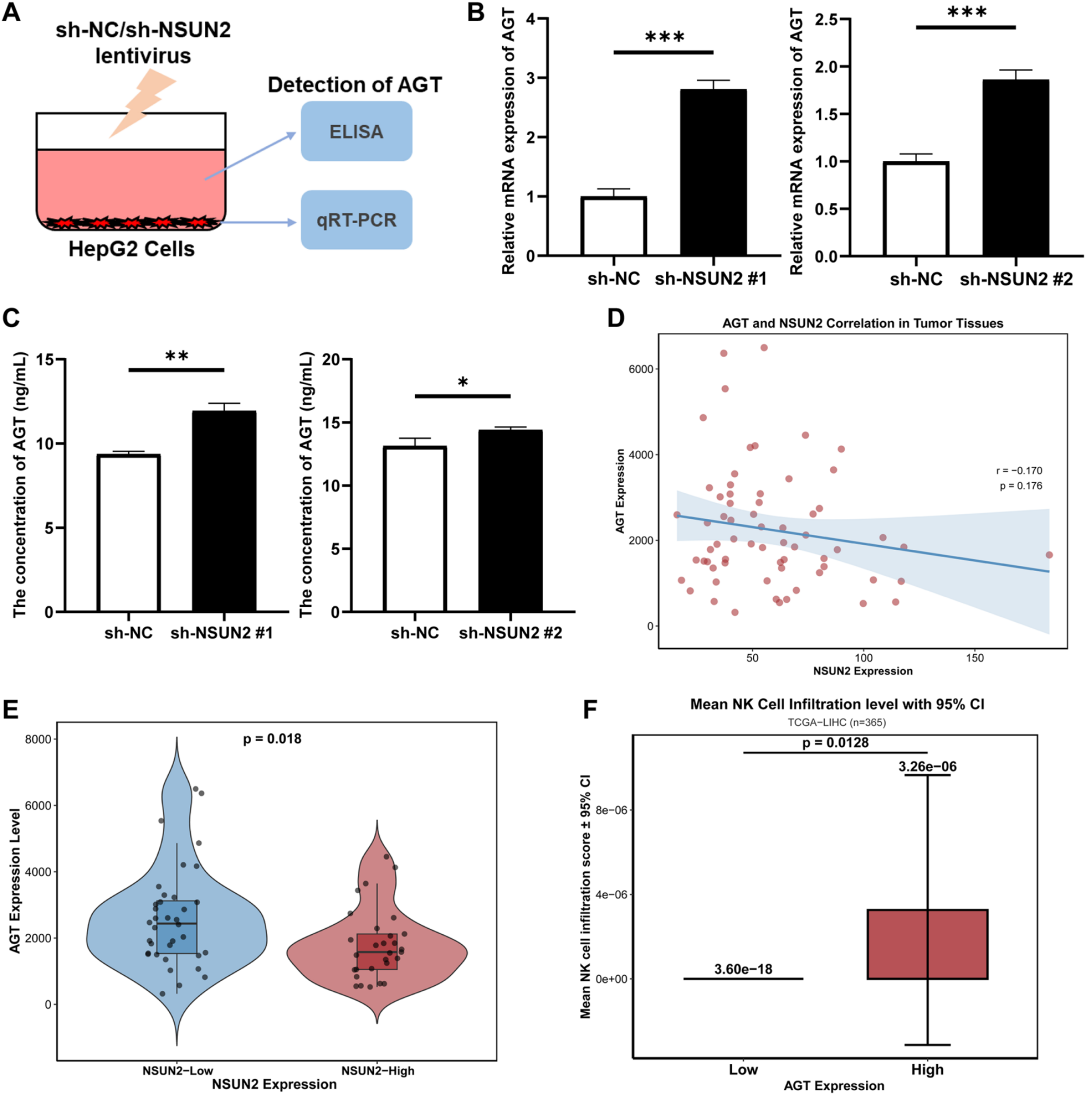
AGT is regulated by NSUN2 and correlates with NK cell infiltration levels in liver cancer. **(A)** Schematic diagram of experimental procedures. **(B)** qRT-PCR assay for detecting AGT expression in sh-NC or sh-NSUN2 (shNSUN2 #1 and #2) HepG2 cells. **(C)** ELISA assay for detecting AGT expression in cell culture supernatants from sh-NC or sh-NSUN2 HepG2 cells. **(D)** Analyzing the correlation between AGT and NSUN2 using the GEO liver cancer cohort. **(E)** Compare the expression levels of AGT between the two cohorts with low and high NSUN2 expression. **(F)** Compare the levels of NK cell infiltration between the two cohorts with low and high AGT expression from TCGA data. Statistical significance was assessed by unpaired t-test or Mann-Whitney U test, depending on sample distribution; *P < 0.05, **P < 0.01, and ***P < 0.001; *N* = 3 independent experiments for **(B, C)**.

To determine the molecular role of AGT in intercellular communication within liver cancer, we examined its expression in sh-NC or sh-NSUN2 HepG2 and JHH-7 cells (**Figure 4A**). The MeRIP-PCR and RNA stability analysis revealed that NSUN2 knock-down decreased AGT mRNA m^5^C modification levels while increasing its stability (**Supplementary Figures 4A, B**). Concurrently, qRT-PCR and ELISA experimental results indicated that both AGT mRNA expression and protein secretion levels in sh-NSUN2 group were significantly elevated compared to the control group (**Figures 4B, C**, **Supplementary Figure 5A**). Analysis using the GEO liver cancer cohort revealed that AGT expression was negatively correlated with NSUN2 expression (**Figure 4D**), particularly in the NSUN2-high expression cohort where AGT expression was significantly lower than in the NSUN2-low expression cohort (**Figure 4E**). Moreover, further analysis of the correlation between AGT expression and NK cell infiltration levels in liver cancer demonstrated that NK cell infiltration level was significantly higher in the AGT high-expression group compared to the AGT low-expression group (**Figure 4F**). In summary, these findings demonstrated that AGT expression level in liver cancer was negatively correlated with m^5^C modification, which may influence tumor progression by regulating NK cell infiltration.

### AGT stimulation enhanced YT-NK cell cytotoxicity

**Figure 5:**
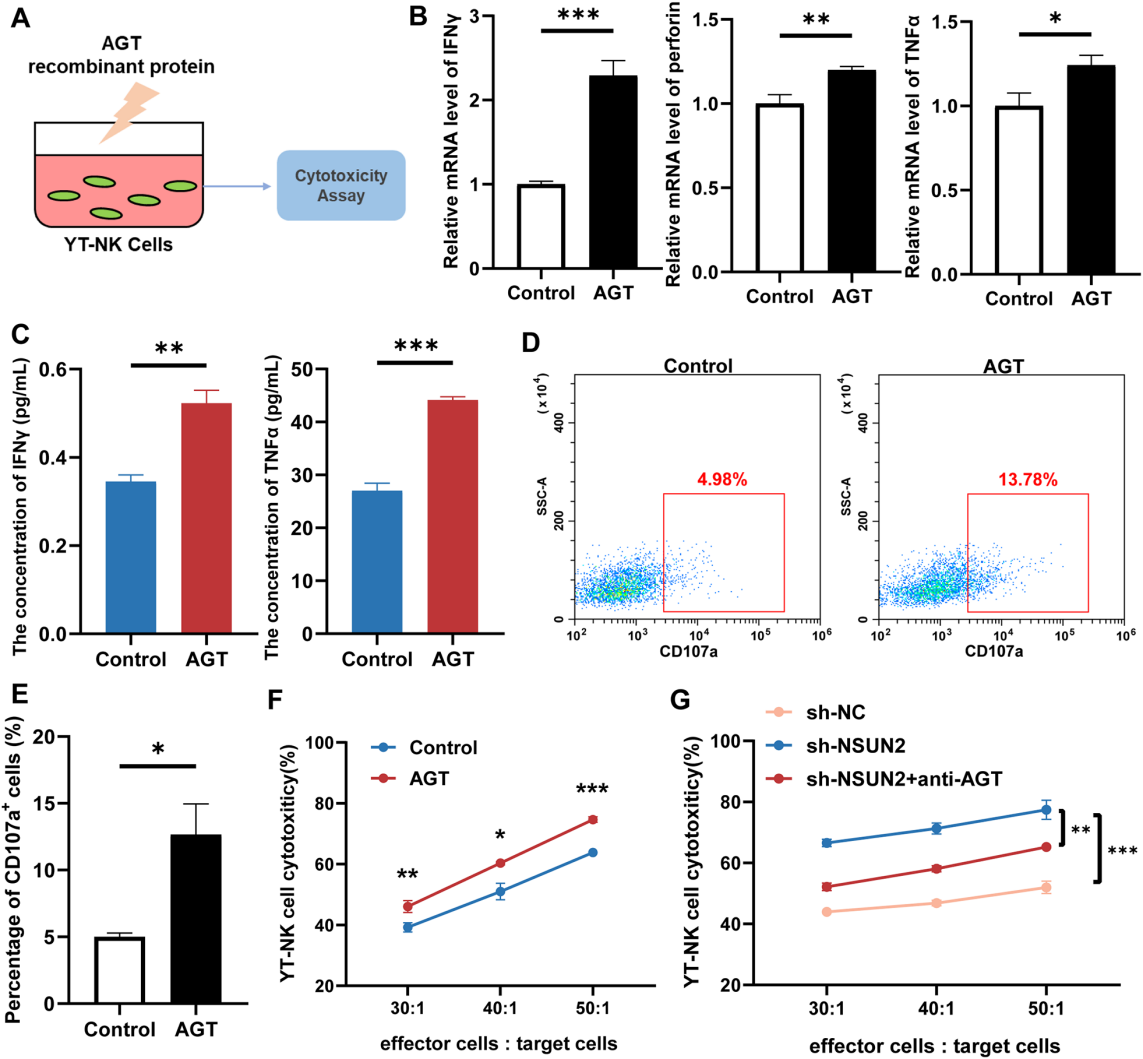
AGT treatment enhances the cytotoxic activity of YT-NK cells. **(A)** Schematic diagram of experimental procedures. **(B)** qRT-PCR assay for detecting the expression of cytotoxic factors in the control group and AGT-treated group. **(C)** ELISA assay for detecting the expression of cytotoxic factors in the control group and AGT-treated group. **(D)** Flow cytometry detection of CD107a^+^ cells. **(E)** Quantitative results for **(D)**. **(F)** Evaluation of NK cytotoxicity by co-culture of YT cells with HepG2 cells, treated with or without AGT. **(G)** Evaluation of NK cytotoxicity by co-culture of YT cells with sh-NC or sh-NSUN2 HepG2 cells or by adding anti-AGT antibodies concurrently. Statistical significance was assessed by unpaired t-test or Mann-Whitney U test, depending on sample distribution; *P < 0.05, **P < 0.01, and ***P < 0.001; *N* = 3 independent experiments for panels **(B–G)**.

To investigate the effects of AGT on NK cell function, the NK cell line YT cells were treated with recombinant AGT protein (**Figure 5A**). The qRT-PCR and ELISA analysis demonstrated that following AGT treatment, the expression levels of NK cell cytotoxicity-related factors, such as IFN-γ, perforin and TNF-α, were significantly elevated compared to the control group (**Figures 5B, C**). Flow cytometry analysis revealed an increased proportion of CD107a^+^ YT cells following AGT treatment, indicating enhanced degranulation activity and toxicity (**Figures 5D, E**). Co-culturing AGT-treated YT cells with HepG2 or JHH-7 cells significantly enhanced the cytotoxic activity of YT cells compared to the control group (**Figure 5F**, **Supplementary Figure 5B**). Furthermore, co-culturing YT cells with sh-NSUN2 HepG2 or JHH-7 cells also markedly increased the killing capacity of YT cells. As expected, adding anti-AGT antibodies into co-culture significantly neutralized the killing capacity of YT cells (**Figure 5G**, **Supplementary Figure 5C**).

### Stimulation with AGT enhanced PBMC-NK cell cytotoxicity

**Figure 6:**
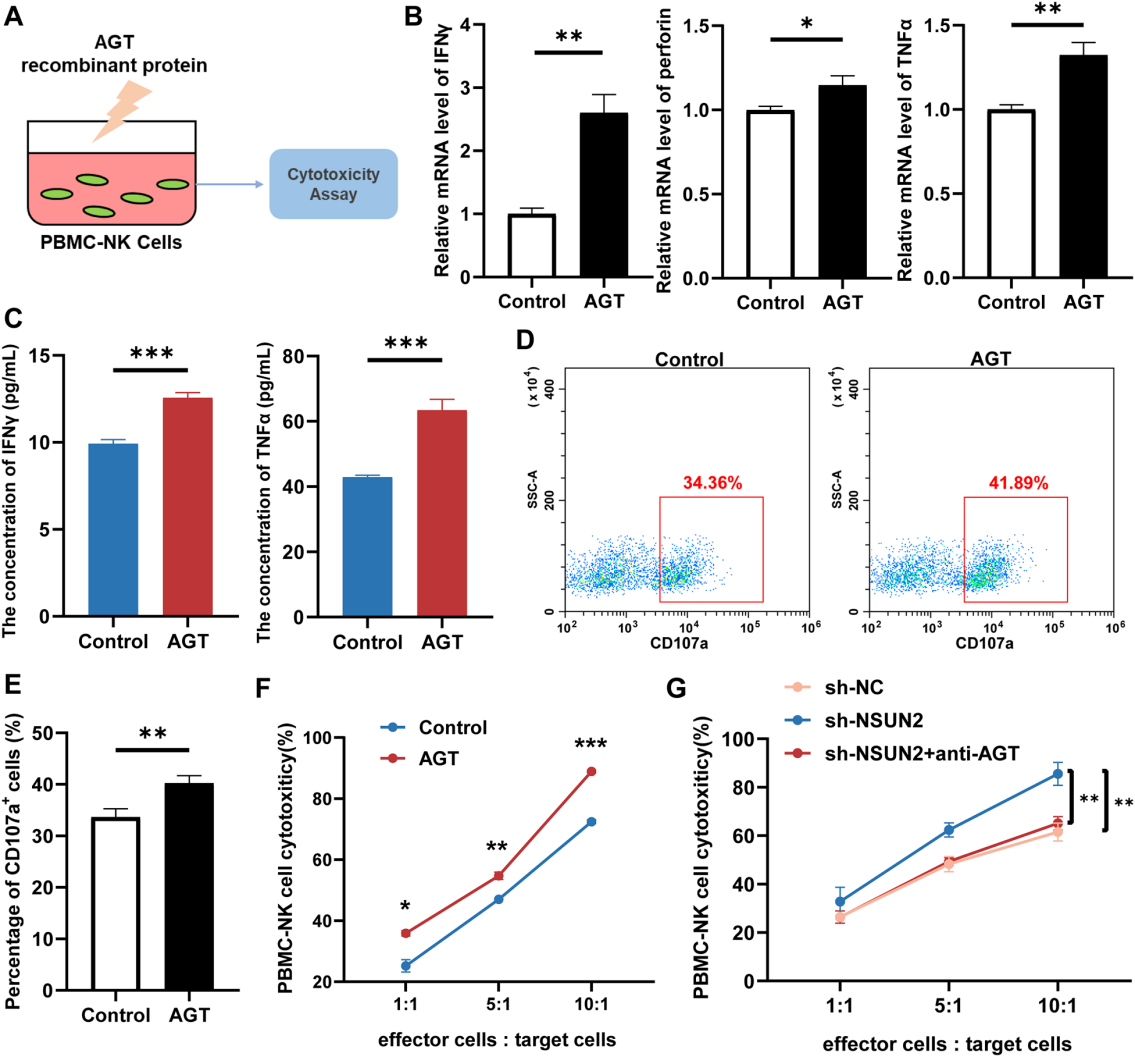
AGT treatment enhances the cytotoxic activity of PBMC-NK cells. **(A)** Schematic diagram of experimental procedures. **(B)** qRT-PCR assay for detecting the expression of cytotoxic factors in the control group and AGT-treated group. **(C)** ELISA assay for detecting the expression of cytotoxic factors in the control group and AGT-treated group. **(D)** Flow cytometry detection of CD107a^+^ cells. **(E)** Quantitative results for **(D)**. **(F)** Evaluation of NK cytotoxicity by co-culture of NK cells with HepG2 cells, treated with or without AGT. **(G)** Evaluation of NK cytotoxicity by co-culture of NK cells with sh-NC or sh-NSUN2 HepG2 cells or by adding anti-AGT antibodies concurrently. Statistical significance was assessed by unpaired t-test or Mann-Whitney U test, depending on sample distribution; *P < 0.05, **P < 0.01, and ***P < 0.001; *N* = 3 independent experiments for panels **(B–G)**.

To further validate the effects of AGT on NK cell function, we treated PBMC-derived NK cells with recombinant AGT protein (**Figure 6A**). Analysis for qRT-PCR, ELISA and flow cytometry demonstrated that PBMC-NK cells exhibited significantly elevated levels and capacity for secreting cytotoxic factors post AGT treatment (**Figure 6B–E**). Co-culturing AGT-treated PBMC-NK cells with HepG2 or JHH-7 cells significantly enhanced the cytotoxic activity of NK cells compared to the control group (**Figure 6F**, **Supplementary Figure 5D**). Moreover, co-culturing PBMC-NK cells with sh-NSUN2 HepG2 or JHH-7 cells also markedly increased the killing capacity of NK cells. Adding anti-AGT antibodies into co-culture significantly neutralized the killing capacity of NK cells (**Figure 6G**, **Supplementary Figure 5E**). Additionally, the co-administration of losartan and AGT did not affect the cytotoxic activity of NK cells against liver cancer cells (**Supplementary Figure 6**). The above results collectively suggested that AGT treatment augmented the cytotoxic activity of NK cells against tumor cells.

### The co-expression of AGT and NSUN2 served as a prognostic risk factor for liver cancer

**Figure 7:**
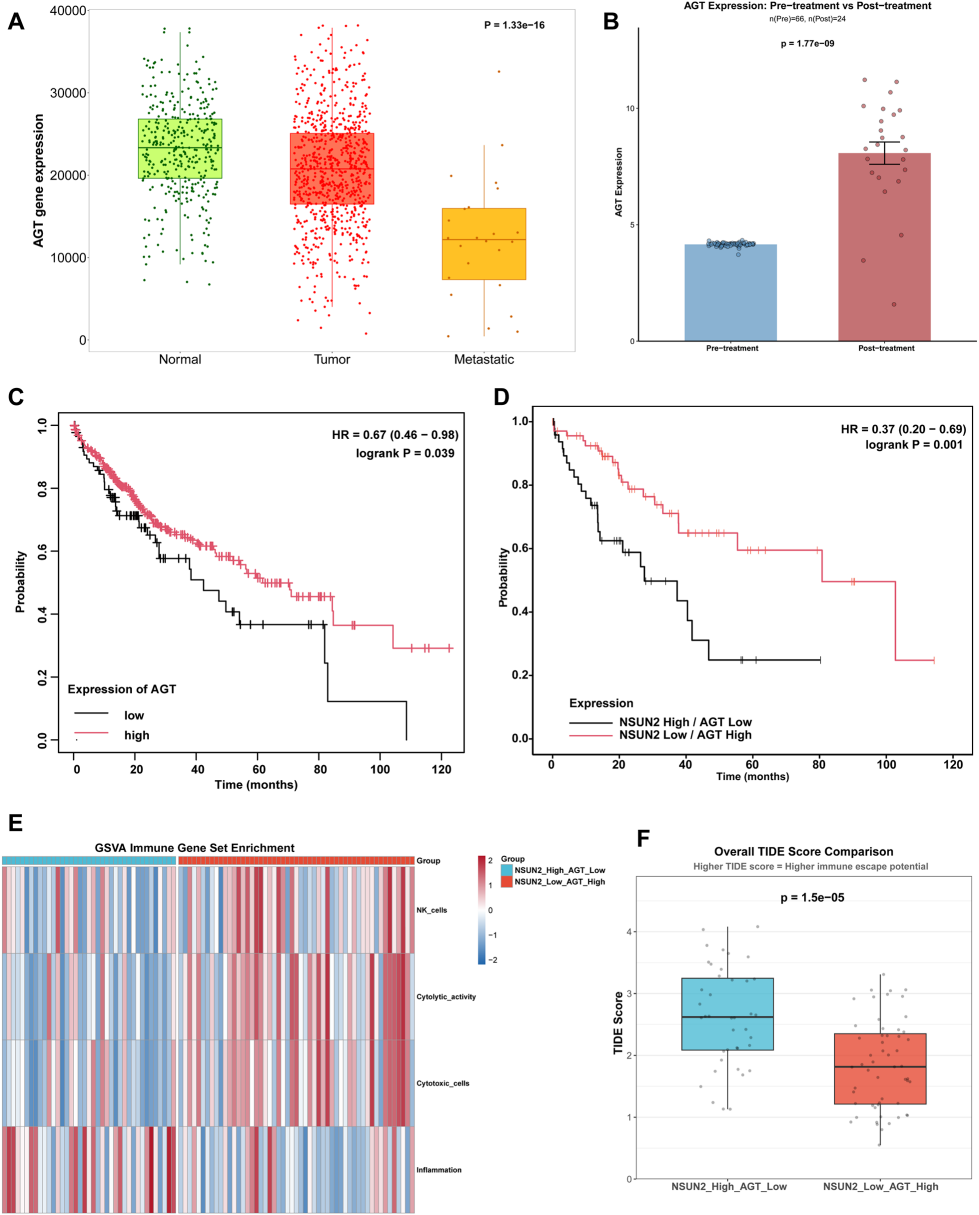
Co-expression of AGT and NSUN2 correlates with prognosis in liver cancer. **(A)** Comparison of AGT expression levels between normal, tumor, and metastatic tissue of liver. **(B)** Comparison of AGT expression levels between the liver cancer patients before and after immunotherapy. **(C)** Survival curves showing the relationship between AGT expression levels and patient overall survival. **(D)** Survival curves showing the relationship between AGT and NSUN2 expression levels and patient overall survival. **(E)** Heatmap showing the enrichment of immune cell gene set signature estimated by GSVA. **(F)** Evaluation of immunotherapy effectiveness based on TIDE score. Statistical significance was assessed by one-way ANOVA **(A)**, Mann-Whitney U test **(B, F)** or log-rank test **(C, D)**.

We conducted a further analysis of the relationship between the expression levels of AGT and NSUN2 and the prognosis in liver cancer using the public patient cohort data. The online tool TNMplot was initially employed to analyze AGT expression changes during liver cancer progression. Results showed that AGT expression levels were significantly reduced in liver tumors and metastatic tissues compared to normal tissues (**Figure 7A**). In the cohort of liver cancer patients before and after anti-PD-L1 therapy, AGT expression levels significantly increased following treatment (**Figure 7B**). Meanwhile, patients with high AGT expression reported markedly improved overall survival rates (**Figure 7C**). Using the TCGA liver cancer cohort data, we classified patients based on AGT and NSUN2 expression levels into two groups: one with high NSUN2 expression and low AGT expression, and the other with low NSUN2 expression and high AGT expression. We observed a significantly higher overall survival rate in the group with low NSUN2 expression and high AGT expression (**Figure 7D**). Furthermore, this group exhibited higher levels of immune cell infiltration and lower potential for immune escape (**Figures 7E, F**). The above-mentioned results showed that the expression levels of AGT and NSUN2 could be used as prognostic indicators for patients with liver cancer.

## Discussion

Elucidating the molecular mechanisms of intercellular communication within the tumor immune microenvironment holds significant promise for the diagnosis and treatment of liver cancer. However, limited research has explored whether m^5^C modification, a key RNA methylation modification, participates in these processes. In this study, we developed GAT-MeRIP, an algorithm for predicting key m^5^C-modified target genes. By integrating multiple transcriptomic sequencing data, we identified AGT as a critical m^5^C-regulated target gene in liver cancer that participates in intercellular communication. Experimental validation demonstrated that AGT treatment enhanced NK cell-mediated killing of liver cancer cells, thereby shaping the tumor immune microenvironment in liver cancer.

RNA modifications refer to post-transcriptional modifications of RNA in both eukaryotic and prokaryotic organisms (26, 27). Over 170 types of RNA modifications have been identified to date, most notably RNA methylation modifications, including m^5^C modifications (27–29). m^5^C modifications can be detected using various biochemical methods, such as bisulfite sequencing, RNA methylation immunoprecipitation sequencing and 5-aza-cytidine interlinking sequencing (30, 31). These detection techniques generally demand high-quality experimental samples and skilled operators, while being time-consuming, labor-intensive, and costly. However, with the increasing volume of sequencing data generated in the post-genomic era, computational algorithms have emerged that enable the computational prediction of m^5^C modification sites (32). These include traditional machine learning-based algorithms like RNAm^5^Cfinder (33) and m^5^CPred-SVM (34), as well as recent deep learning approaches such as Trans-m^5^C (19) and m^5^C-iDeep (20) based on neural networks. However, these algorithms focus solely on predicting m^5^C modification sites. While identifying the presence of m^5^C sites on RNA is important, recognizing functionally significant genes mediated by m^5^C modifications holds greater significance for understanding biological processes in diseases like cancer. Therefore, we developed GAT-MeRIP, a key m^5^C-methylated target gene screening method based on a graph attention neural network model. Using this algorithm, we identified a set of key target genes regulated by m^5^C modification in liver cancer cells. Subsequently, by integrating single-cell transcriptomic data from liver cancer and cell-cell communication analysis, we determined that AGT was a key factor in liver cancer intercellular communication and was regulated by m^5^C modification.

NSUN2 is a well-studied RNA m^5^C methyltransferase that plays a crucial role in the development and progression of liver cancer (35). Shi et al. demonstrated that m^5^C modification of MALAT1 catalyzed by NSUN2 and ALYREF promotes resistance to sorafenib in liver cancer (36). Li et al. reported that NSUN2-mediated SREBP2 m^5^C modification induces proliferation and metastasis of liver cancer cells by promoting cholesterol synthesis (37). However, the role of NSUN2 in regulating the communication between liver cancer cells and immune cells is still not well understood. In this study, we employed the GAT-MeRIP algorithm to integrate multiple transcriptomic sequencing data, identifying NSUN2 as a potential mediator of m^5^C modification in AGT that participates in liver cancer cell-cell communication. Our results further demonstrated a negative correlation between the expression levels of NSUN2 and AGT, and liver cancer patients with low NSUN2 expression and high AGT expression exhibited a better prognosis.

AGT is primarily produced by the liver and is a component of the renin-angiotensin system (RAS) (38). Notably, RAS participates in tumorigenesis and progression, such as promoting tumor angiogenesis and contributing to tumor cell invasion and migration (39–41). Angiogenesis and remodeling are also major characteristics of tumors, playing a crucial role in tumor growth and distant metastasis (42, 43). Previous studies have demonstrated that AGT possesses anti-angiogenic and anti-tumor effects (44, 45). Vincent et al. demonstrated that AGT inhibits angiogenesis and tumor growth in liver cancer by generating transgenic mice overexpressing human AGT (46). However, the precise molecular mechanisms underlying the effects of AGT, particularly its role in intercellular communication within liver cancer, require further elucidation. In our experiments, we revealed a negative correlation between AGT expression and NK cell infiltration in liver cancer. Treatment of NK cells with recombinant AGT increased the secretion of cytotoxic cytokines and enhanced their killing capacity against liver cancer cells. Furthermore, analysis of liver cancer immunotherapy cohorts revealed significantly elevated AGT expression level post-treatment. These findings collectively suggested that AGT may suppress liver cancer progression by enhancing NK cell function. However, our study has certain limitations. First, GAT-MeRIP is a self-supervised model that solely depends on the input experimental methylation profiles but includes no other external labels of gene importance, therefore it will produce false-negative results when the genes are absent in the methylation profiles. Second, the experimental exploration was conducted only at the cellular level and requires further validation in *in vivo* experiments. Third, although our result suggests that typical AGTR are not likely to mediate the NK cell modulation effects, the specific receptor and molecular signaling pathways through which AGT, as a ligand protein, regulates NK cell function remain further investigation.

In summary, we employed the GAT-MeRIP method for screening key m^5^C-methylated target genes. By integrating multiple transcriptomic sequencing data analyses and experimental validation, we revealed that AGT, an m^5^C-modified secreted protein found in liver cancer cells, influenced cancer progression by modulating NK cell function. This finding contributes to understanding the molecular mechanisms of intercellular communication in liver cancer and lays the groundwork for developing targeted immunotherapeutic agents against the disease.

## Data Availability

The datasets presented in this study can be found in online repositories. The names of the repository/repositories and accession number(s) can be found below: https://ngdc.cncb.ac.cn/gsa-human, HRA014706.
