# Supplementary material for: Deep learning model and omics screening highlight angiotensinogen as a 5-methylcytosine (m5C) regulated mediator of tumor-microenvironment communication in liver cancer
Source: Front Immunol. 2026 Feb 20;17:1752802. doi: 10.3389/fimmu.2026.1752802 (PMC12963822; doi:10.3389/fimmu.2026.1752802)
Supplement: Supplementary file 1 [file DataSheet1.pdf]

Supplementary Material for

Deep Learning Model and Omics Screening Highlight Angiotensinogen as a 5-Methylcytosine (m^5C)-Regulated Mediator of Tumor-Microenvironment Communication in Liver Cancer

Supplementary Figure

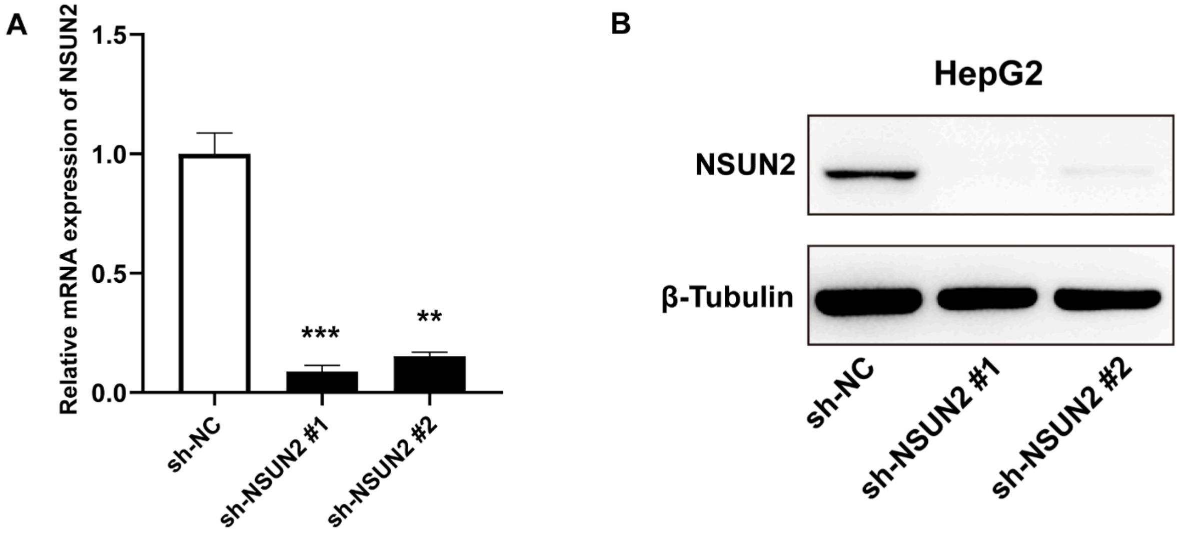

Supplementary Figure 1. Analysis for the level of NSUN2 expression in HepG2 cells following NSUN2 knock-down. (A) qRT-PCR assay for mRNA expression of NSUN2 after transfection of lentiviral interference fragments. (B) Western blot assay for protein expression of NSUN2 after transfection of lentiviral interference fragments.

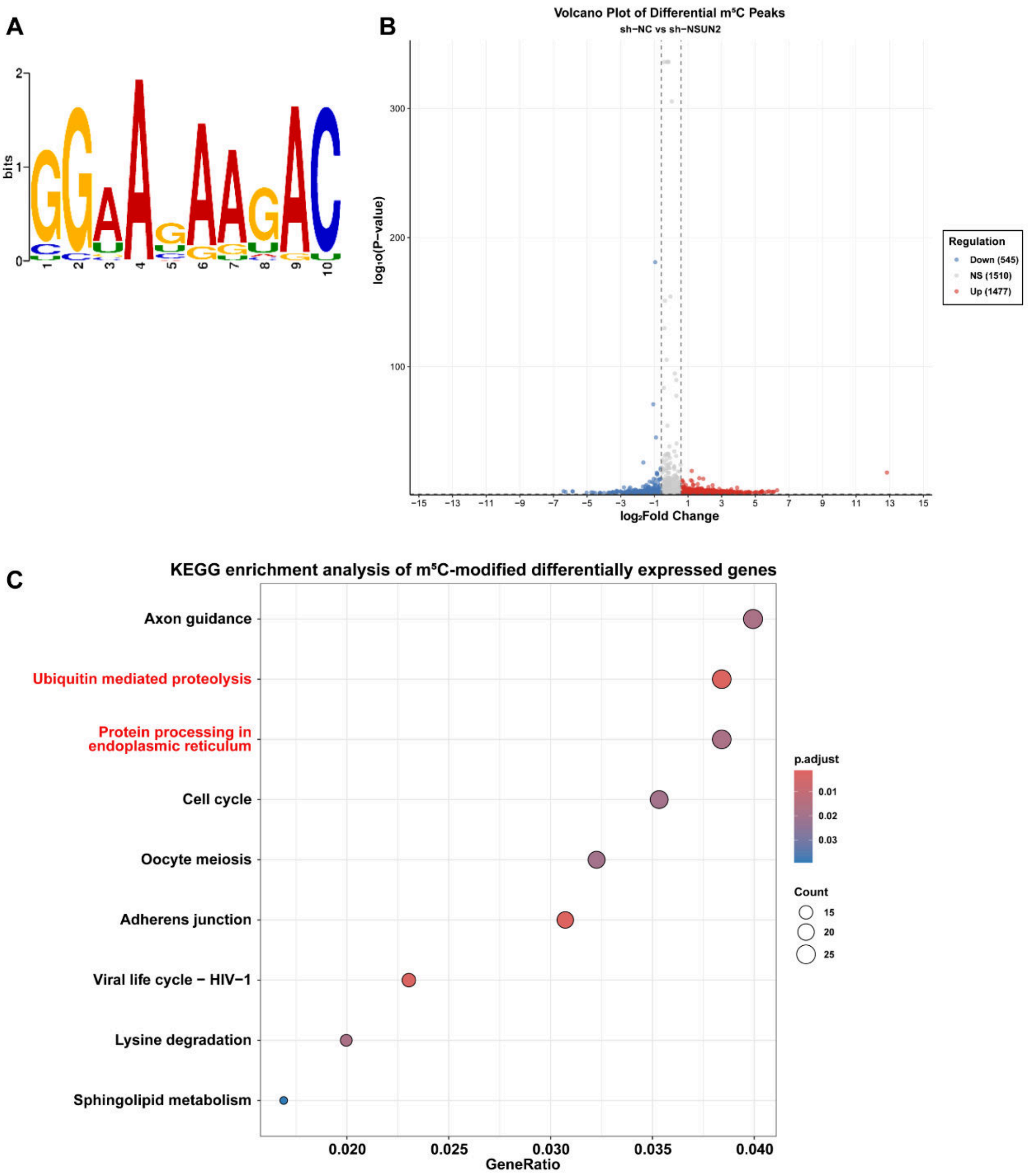

Supplementary Figure 2. The m^{5}C-MeRIP-seq data analysis from HepG2 cells following NSUN2 knock-down. (A) The consensus motif identified from the m^{5}C methylation peaks. (B) Volcano plot depicting the differential m^{5}C peaks. In comparison with sh-NSUN2 cells, sh-NC cells show more up-regulated methylation peaks than down-regulated methylation peaks, indicating higher m^{5}C methylation activity in sh-NC than sh-NSUN2 cells. (C) KEGG enrichment analysis of m^{5}C-modified differentially expressed genes.

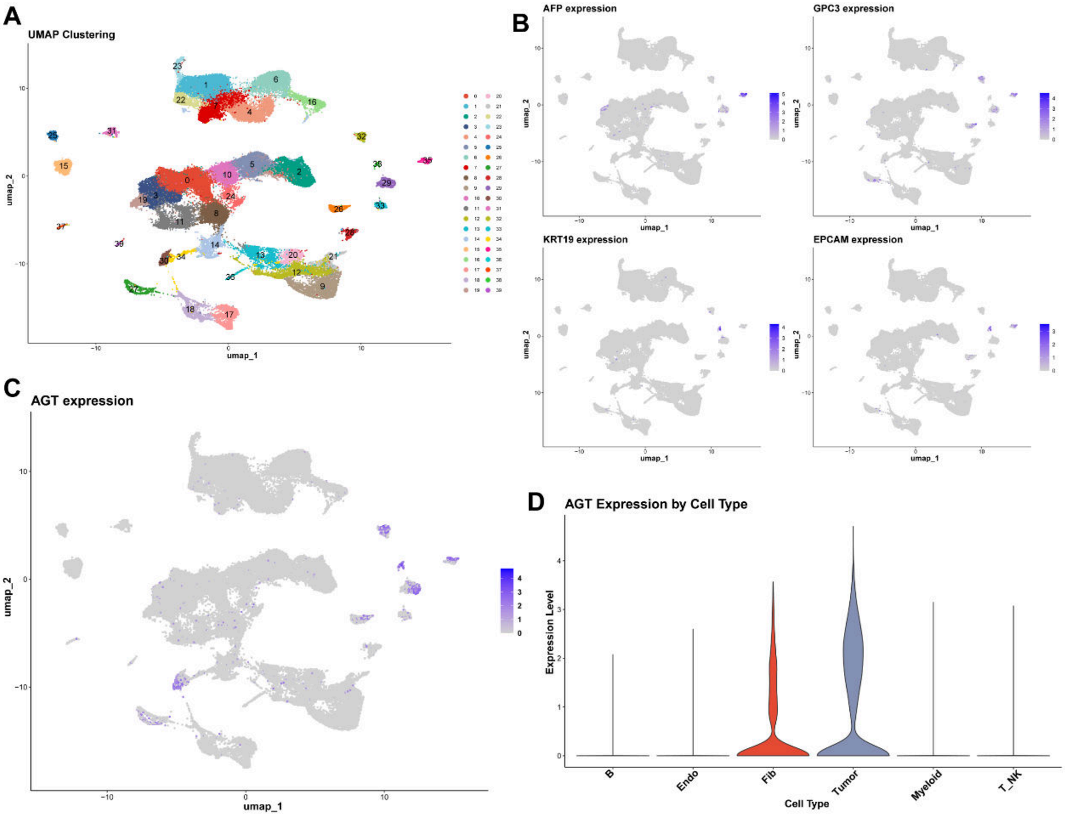

Supplementary Figure 3. The scRNA-seq data analysis of liver cancer. (A) UMAP plot of cell clusters. (B) UMAP plot showing expression of marker genes for tumor cells. (C) UMAP plot showing the distribution of AGT expression. (D) Compassion of expression levels of AGT between different cell groups.

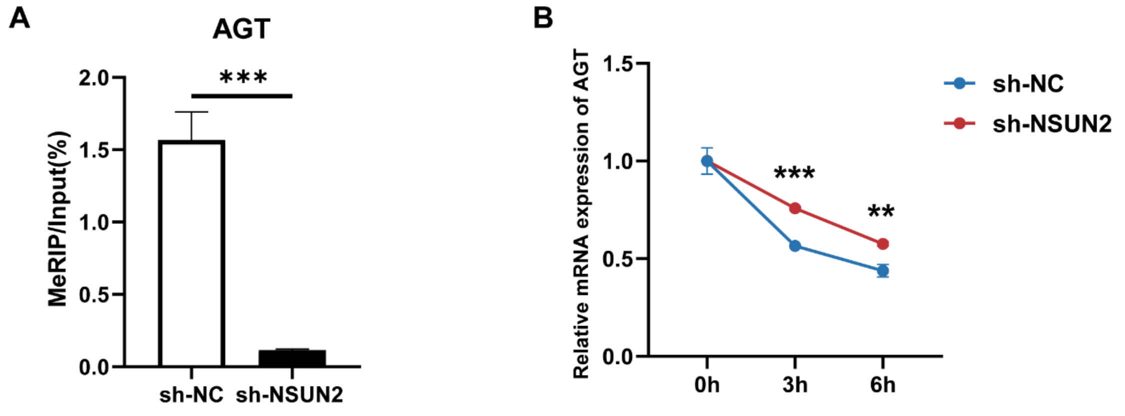

Supplementary Figure 4. Detection of m^{5}C modification and stability of AGT mRNA following NSUN2 knock-down. (A) MeRIP-PCR assay for detecting m^{5}C modification levels in AGT mRNA. (B) Actinomycin D assay of AGT mRNA stability. Statistical significance was assessed by unpaired t-test or Mann-Whitney U test, depending on sample distribution; $**P < 0.01$, and $***P < 0.001$; $N = 3$ independent experiments for panels A and B.

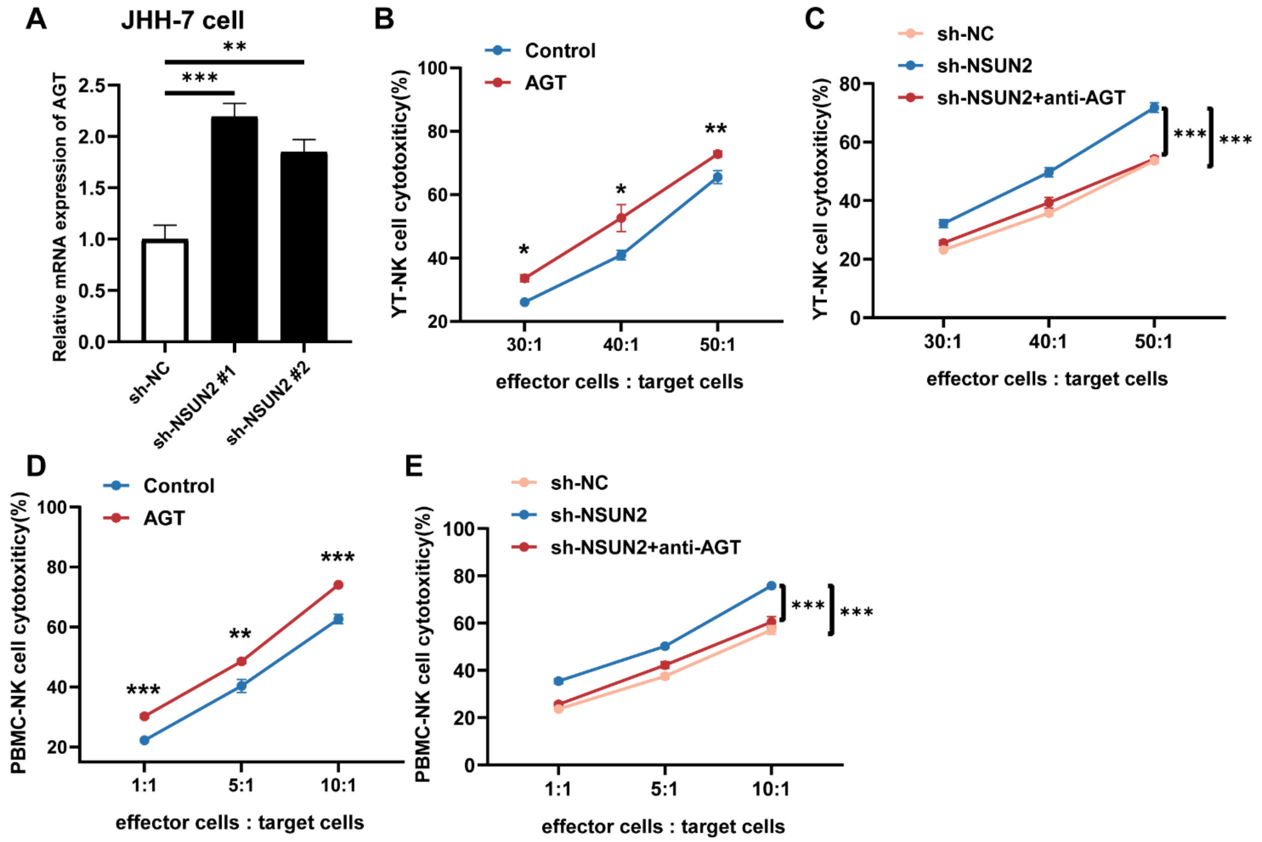

Supplementary Figure 5. Co-culture assay for detecting NK cell cytotoxicity. (A) qRT-PCR assay for detecting AGT expression in sh-NC or sh-NSUN2 (shNSUN2 #1 and #2) JHH-7 cells. (B and D) Evaluation of NK cytotoxicity by co-culture of YT-NK cells (B) and PBMC-NK cells (D) with JHH-7 cells, treated with or without AGT. (C and E) Evaluation of NK cytotoxicity by co-culture of YT-NK cells (C) and PBMC-NK cells (E) with sh-NC or sh-NSUN2 JHH-7 cells or by adding anti-AGT antibodies concurrently. Statistical significance was assessed by unpaired t-test or Mann-Whitney U test, depending on sample distribution; * $P < 0.05$, ** $P < 0.01$, and *** $P < 0.001$; $N = 3$ independent experiments for panels A-E.

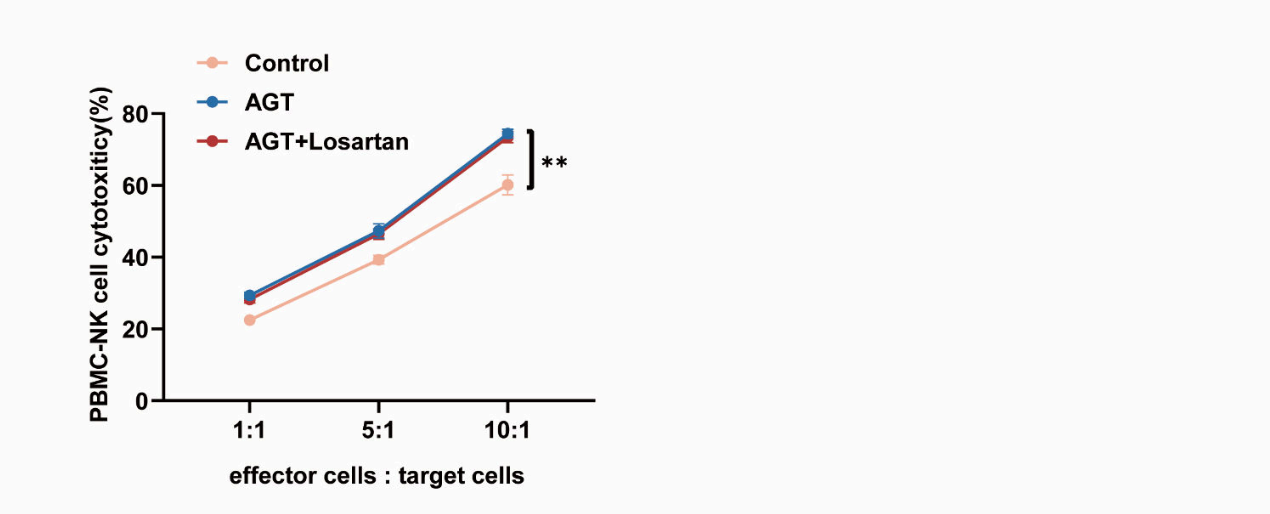

Supplementary Figure 6. Evaluation of NK cytotoxicity by co-culture of NK cells with HepG2 cells, treated with or without AGT and losartan. Statistical significance was assessed by unpaired t-test. ***P* < 0.01; *N* = 3 independent experiments.

Supplementary Table

Supplementary Table 1. Primers required for qRT-PCR

Genes	Sequence
β-Actin	Forward: CACCATTGGCAATGAGCGGTTC<br>Reverse: AGGTCTTTGCGGATGTCCACGT
NSUN2	Forward: ACCTGGCTCAAAGACCACACAG<br>Reverse: TGGCTTGATGGACGAGCAGGTA
Interferon gamma	Forward: GAGTGTGGAGACCATCAAGGAAG<br>Reverse: TGCTTTGCGTTGGACATTCAAGTC
TNF alpha	Forward: CTCTTCTGCCTGCTGCACTTTG<br>Reverse: ATGGGCTACAGGCTTGTCACTC
Perforin	Forward: ACTCACAGGCAGCCAACTTTGC<br>Reverse: CTCTTGAAGTCAGGGTGCAGCG
AGT	Forward: CATTCTTCCTCTCCTCCTT<br>Reverse: TCTACCAGTCCTCTATCCA

Supplementary Table 2. Key m^{5}C-modified target genes were identified from the m^{5}C-MeRIP-seq data using GAT-MeRIP

Genes	Importance score
RN7SL2	1.000
RN7SL3	1.000
TMEM123	1.000
COL27A1	1.000
GPX3	1.000
HNRNPA1P10	1.000
SNHG7	0.999
SERPINF1	0.998
C1QTNF3	0.997
BOK	0.996
AGT	0.996
COLEC11	0.995
PRAP1	0.995
PLXNC1	0.994
ERN1	0.992
H6PD	0.991
ST3GAL2	0.991
SLC40A1	0.989
UGGT2	0.989
EIF2AK3	0.988
METTL21A	0.988
OTUD3	0.985
CCNJL	0.984
LRRC20	0.983
B3GAT2	0.980
MROH8	0.980
ADM2	0.978
TARBP1	0.977
DNAJB11	0.967
SLC25A5-AS1	0.967
TTC13	0.966

TNRC18	0.963
PAQR8	0.962
RNF114	0.957
DNAJC3	0.954
VWCE	0.954
PDIA6	0.945
TMTC3	0.944
LOX	0.944
PIGK	0.940
TTC17	0.939
TEX261	0.938
IDI1	0.938
SDF2	0.935
CASP7	0.934
CRTC3	0.926
TP53INP2	0.920
ST6GALNAC6	0.917
DEXI	0.917
DNASE1L1	0.911
GSR	0.910
HPS1	0.908
PODXL	0.907
PAWR	0.906
ERLEC1	0.902
PTPRN2	0.892
SPON2	0.892
MET	0.892
EPS15L1	0.891
FKBP10	0.888
ERLIN2	0.883
TXNDC5	0.883
RUSC1-AS1	0.879
ITM2C	0.878

GJB1	0.875
CGNL1	0.871
GCHFR	0.861
PRPSAP2	0.861
LAMB1	0.856
ERP29	0.850
APOA2	0.849
BPIFB2	0.849
CNN3	0.834
IL6R	0.821
ANKRD65	0.815
CLU	0.813
KIAA1217	0.813
KCTD5	0.811
P4HB	0.806
CKAP2	0.806
SPATA21	0.805
SOX18	0.803
ATP1B1	0.803
DNAJC16	0.798
APOE	0.798
STMN1	0.793
SERHL	0.788
GLRX3	0.786
HSPA9	0.782
COLGALT1	0.780
DNAJC17	0.777
HAUS2	0.775
BEST1	0.773
HNRNPH3	0.772
WDR11	0.771
RSF1	0.768
OCEL1	0.768

SPCS3	0.763
MYL6B	0.763
LPP	0.762
POTEE	0.760
PLOD3	0.759
CHRAC1	0.758
UQCRB	0.757
GOLGA4	0.754
PRKAR2A	0.754
ZC3H13	0.753
SLC35C1	0.753
FLNB	0.747
P3H1	0.742
INSR	0.741
GAA	0.740
NUP210	0.735
SPCS2	0.732
TBC1D13	0.732
NECAB3	0.731
PHIP	0.730
CABP7	0.730
ARPC5L	0.730
CSE1L	0.726
AP1M1	0.723
PAQR7	0.722
APOB	0.721
CDKN2AIP	0.721
HNRNPA1L2	0.718
MAST1	0.716
COPS8	0.712
TMOD3	0.709
PPIB	0.708
HELLS	0.707

SRRM5	0.706
FNDC3B	0.703
UGGT1	0.700
STT3A	0.699
SDHAP1	0.695
GNAS	0.695
EMC7	0.694
LOC100287015	0.692
ENOSF1	0.691
TUBB2B	0.689
SHC1	0.689
CALR	0.688
TOR1AIP1	0.685
SUN2	0.685
PCDHB2	0.684
PLOD2	0.683
DSP	0.679
GGTLC2	0.678
SLC5A10	0.677
CLASP2	0.677
SNRPD1	0.676
BCAP31	0.674
ASPH	0.673
SYVN1	0.672
MCM3AP-AS1	0.667
CAP1	0.665
GAB2	0.665
PPP3CB-AS1	0.663
MAFIP	0.655
POU2F1	0.653
NCKAP1	0.650
EIF3D	0.648
RAB10	0.648

C12orf76	0.648
STAG1	0.647
PRPS1	0.645
HOXA-AS2	0.645
SUPT6H	0.644
DNAJB12	0.644
SPTBN1	0.643
SNRPD3	0.642
MOGS	0.638
RPS6KA3	0.633
TMED2	0.632
LUZP1	0.631
EIF4G2	0.629
RPN1	0.628
LRPAP1	0.627
DNAJC1	0.626
MPRIP	0.621
CEP76	0.619
NME1	0.618
HNRNPUL2	0.617
PLA2G2E	0.617
PLOD1	0.616
SEC22C	0.615
DNAJC19	0.613
HNRNPA3	0.613
ZNF814	0.611
WTAP	0.611
TCFL5	0.610
AGAP7P	0.610
SLC26A10	0.608
SERPINH1	0.607
WDR6	0.607
UBXN1	0.604

PAK2	0.602
RANBP1	0.601
PGF	0.600
CPEB4	0.599
SNORD3A	0.597
SREBF2	0.596
RPLP1	0.593
PPP6C	0.592
HNRNPF	0.592
SPTAN1	0.591
SLC25A27	0.588
FBXO25	0.588
RNF40	0.588
SMLR1	0.587
SDCBP2-AS1	0.586
ERBB2	0.586
SEL1L	0.586
HS3ST3A1	0.586
GNAI3	0.585
EPS15	0.585
SLC25A43	0.583
RAB1B	0.579
STAG3L1	0.579
PPP2R2A	0.579
DNAJC7	0.579
TMCO4	0.576
LSM4	0.575
AHCYL1	0.575
HNRNPH2	0.574
NIPA1	0.573
TJP2	0.573
TMC8	0.572
NAALADL1	0.569

STK4	0.568
MYL6	0.568
SVIL	0.568
LRPPRC	0.567
TOPBP1	0.567
SNW1	0.566
SLC35D1	0.564
SUZ12P1	0.564
KLC1	0.563
USP20	0.562
CARM1	0.561
TREH	0.561
NUDT15	0.560
TMEM150B	0.559
MGAT4A	0.558
ASAH2	0.558
FAM131A	0.557
CCDC144NL-AS1	0.557
PRDX3	0.556
ASAH2B	0.556
GPS1	0.556
ZNF79	0.555
SAMD8	0.554
ZSCAN16-AS1	0.554
SNN	0.554
LGR4	0.553
TMED10	0.553
GANAB	0.552
RPL32P3	0.549
OTUD4	0.548
KPNB1	0.547
PACS2	0.546
IPO7	0.546

RASA4B	0.544
C20orf194	0.544
EIF4E2	0.543
GPR157	0.541
GPR137	0.540
PMS2CL	0.539
INPPL1	0.538
FAM229A	0.537
RASA4	0.537
ZNF138	0.535
CSDE1	0.534
VCL	0.533
ABHD13	0.533
TCTN2	0.533
SLC1A7	0.532
SLC25A3	0.532
ZNF266	0.532
GOLGA2P5	0.532
PRTG	0.531
TCAIM	0.531
CHST9	0.530
BMP8A	0.530
MTRNR2L8	0.530
LOC389906	0.528
TUBB3	0.527
ELAC1	0.526
KIAA0895L	0.525
YPEL2	0.525
LDHA	0.525
FTH1P3	0.525
DDX5	0.524
ARPC1B	0.524
ZNF443	0.523

PSMC5	0.523
TNFRSF11A	0.522
SCAND2P	0.522
ZNF789	0.522
PSMA3-AS1	0.522
PPP2R5A	0.520
SNRPB	0.519
ARPC4	0.519
GLUD1P3	0.519
TUG1	0.519
CYB5R3	0.518
SEMA3G	0.518
TPM3	0.518
SNORD18C	0.517
OR51B5	0.515
HSPA8	0.515
MAP2K2	0.514
COPA	0.514
RNF130	0.514
PLEC	0.513
UBA52	0.512
PRKDC	0.511
HDAC6	0.511
PRKAR1A	0.509
ASXL2	0.508
ATP6V0E2	0.508
TUBA1C	0.507
CD164	0.507
ZNF473	0.506
YBX1	0.505
ASXL1	0.505
NPNT	0.504
PXN	0.502

RTN4	0.502
HNRNPUL1	0.500
STRAP	0.498
COPS2	0.498
ESPL1	0.496
TXN	0.496
DLST	0.496
LGALS3BP	0.494
ARRB1	0.492
CYB561A3	0.490
PHB	0.490
RPL34	0.487
AHNAK	0.485
KRT18	0.485
RNF44	0.484
HADHB	0.484
ZNF202	0.483
RPL29	0.483
NRSN2	0.482
HNRNPDL	0.481
HCFC1	0.481
CTNNA1	0.480
SKI	0.480
BSG	0.480
HNRNPU	0.480
FITM2	0.479
ALG8	0.479
SSR1	0.479
KRT8	0.476
LIMA1	0.474
ALG10	0.473
JKAMP	0.473
SETD1A	0.472

ZP3	0.471
SLC17A5	0.471
AKT1	0.470
ATXN1	0.469
UQCRHL	0.468
RPL8	0.468
RHBG	0.468
CLDN12	0.468
HADHA	0.467
TM2D2	0.467
SPDL1	0.467
RPL17	0.467
TMTC4	0.467
TFRC	0.466
LMAN1	0.466
TRAP1	0.464
ITGB1	0.464
TMEM243	0.464
PSMA2	0.464
TMEM37	0.464
SEC62	0.464
PPP2R5E	0.463
MFSD8	0.462
AP5M1	0.462
FOXO1	0.462
SLC35F5	0.461
LRFN3	0.461
AIFM2	0.461
ANKMY1	0.460
SLC8B1	0.458
RAB7A	0.458
SFPQ	0.457
LRRC3	0.457

TMEM181	0.456
DESI2	0.456
GHDC	0.456
CDKN1A	0.455
LCMT1	0.455
PSME3	0.455
ATP1A1	0.455
PSMC2	0.455
RPS10	0.455
SEMA4F	0.454
ONECUT2	0.454
HERPUD2	0.453
NPIPB11	0.453
PKIB	0.452
PSMD11	0.451
SHMT2	0.451
SP5	0.451
ZDHHC9	0.450
CNNM4	0.450
RPL38	0.450
G6PC	0.450
HSP90AB1	0.449
MANEAL	0.449
NIPA2	0.449
RPS20	0.449
COMMD7	0.448
SNX13	0.447
CDC5L	0.447
CFL1	0.447
NRP2	0.447
ASH2L	0.446
GRINA	0.446
CDH1	0.445

TSPAN17	0.445
EP300	0.444
CLDND2	0.444
LARP1	0.444
MST1L	0.443
SOD1	0.443
TRAM2	0.442
DYNLL1	0.442
PHF13	0.442
SUPT5H	0.442
SLC44A5	0.441
LAT2	0.441
IER5L	0.441
RNF43	0.441
VDAC1	0.440
GSAP	0.439
PRKCZ	0.439
CDC73	0.439
RPS27	0.438
SSB	0.438
NPM1	0.438
CNNM2	0.438
SNRNP200	0.437
PABPC4	0.437
SMAD4	0.437
SNORD3C	0.436
ATP8B2	0.436
YAP1	0.435
NUDCD2	0.435
COMMD2	0.434
ERMP1	0.434
DLX1	0.434
SCAMP5	0.433

TMEM164	0.433
ASB13	0.433
RBBP8NL	0.433
FAM20B	0.433
IGF1R	0.432
PPP2R1A	0.432
PRR14L	0.432
AP5S1	0.432
PEX26	0.432
RPL28	0.431
C4orf3	0.430
SLC35E3	0.429
FAM120AOS	0.429
NCL	0.429
MMP15	0.428
CROCCP3	0.427
CSK	0.427
MMD	0.427
HEG1	0.426
SIDT2	0.426
TMEM120B	0.426
WWP2	0.425
COMMD5	0.425
DFFB	0.425
BRI3	0.425
FAAH	0.425
ALG11	0.425
INTS9	0.424
TPRA1	0.424
SLC43A1	0.423
ADO	0.423
PHTF1	0.423
PDZD7	0.423

TMEM242	0.422
FAM222A	0.422
CLSTN3	0.422
YIPF4	0.422
TUBA1A	0.422
TSKU	0.421
RBM24	0.421
CILP2	0.420
FBXO7	0.420
FAM122A	0.420
SHROOM1	0.420
PEX11B	0.419
AKR1C4	0.418
FUT11	0.418
EXTL2	0.418
YIPF6	0.418
ARIH2	0.418
ATRN	0.417
SLC23A2	0.417
UBR5	0.417
GRAP	0.417
MAL2	0.417
DAGLA	0.417
SLC16A13	0.417
TP53I13	0.416
GCNT4	0.416
TSPAN31	0.416
OTUB1	0.415
ZFYVE26	0.415
PIGO	0.414
ATP13A3	0.413
SPG11	0.413
PTP4A2	0.413

ZNF410	0.412
TRAPPC6B	0.412
MYPOP	0.412
ITFG2	0.412
PBX3	0.412
TECPR1	0.412
IFT46	0.411
ZDHHC24	0.411
TMEM186	0.411
HUWE1	0.410
MFSD10	0.410
PPP2R1B	0.410
KRIT1	0.410
ANKLE2	0.410
FAM122C	0.410
STRN4	0.410
TMEM68	0.410
FECH	0.410
TMEM245	0.410
NXPE3	0.410
ACVR2A	0.410
SERTAD2	0.409
BAG2	0.409
C6orf120	0.409
LAPTM4B	0.409
CYP2S1	0.409
ATRAID	0.408
COMMD4	0.408
HIC2	0.408
STRIP1	0.408
STARD7	0.408
SLC3A1	0.407
CTTNBP2NL	0.407

TNIP1	0.407
SNX14	0.407
METTL14	0.407
ZNF587	0.407
MOB4	0.406
TICRR	0.406
MTRR	0.406
HLF	0.405
ZNF580	0.405
TMEM11	0.405
NRARP	0.405
EXT1	0.405
RPS16	0.405
SLC29A2	0.405
SSTR5	0.405
SLMAP	0.404
DTD2	0.404
TMEM45B	0.404
MZT2B	0.404
SRGAP2B	0.404
SLC39A1	0.404
MYRF	0.403
NEU3	0.403
ABCA2	0.403
ZNF331	0.403
TTC30A	0.402
CCDC91	0.402
TMEM63A	0.402
RPS12	0.402
GGT7	0.402
PTGFRN	0.402
LRP10	0.401
ZDHHC12	0.401

ADIPOR1	0.401
TMBIM6	0.401
ZNF226	0.401
ORMDL3	0.401
BSDC1	0.401
NAT9	0.401
CSGALNACT2	0.401
TRAPPC1	0.401
YWHAB	0.400
TMEM30A	0.400
MATR3	0.400
NBPF19	0.400
RPS3	0.399
BNIP3	0.399
ADCY6	0.399
C7orf26	0.399
APH1A	0.399
INTS7	0.398
MIDN	0.398
LMAN2L	0.398
VTI1A	0.398
NDUFC2	0.398
THEM6	0.398
GPR108	0.397
ATP9A	0.397
DPEP1	0.397
C12orf75	0.397
HMGB1	0.396
VAPA	0.396
MVB12B	0.396
ANO9	0.396
DLX2	0.396
CXCL16	0.396

DOC2A	0.396
CCDC8	0.396
C5orf24	0.395
CDS1	0.395
TTC21B	0.395
TSC1	0.395
PGAP1	0.395
TRIM16L	0.395
SMURF1	0.394
SERINC1	0.394
INO80C	0.394
MEGF8	0.394
SNX22	0.394
SP140L	0.393
GSTA1	0.393
CBX3	0.393
FAM160A2	0.393
ZNF140	0.393
SLC11A2	0.393
FAM13B	0.392
MAN2B2	0.392
SMPD1	0.392
FAM102A	0.392
C16orf58	0.392
ACVR2B	0.392
EXOC2	0.392
GAPDH	0.391
P2RX4	0.391
CD63	0.391
SIAE	0.391
CREB3L2	0.391
DRAM1	0.391
CEP350	0.390

SLC29A1	0.390
LYRM1	0.390
COMMD1	0.390
ENTPD4	0.390
SERAC1	0.389
LRRC8B	0.389
NAPB	0.389
INO80E	0.389
TJAP1	0.389
ADAMTSL4	0.388
STX16	0.388
CNPPD1	0.388
ATP11A	0.388
ESPN	0.388
FN1	0.388
RPL12	0.388
SFN	0.388
RRAGB	0.388
NR1H2	0.388
ATP2A3	0.388
CYP4F11	0.387
ZBED5	0.387
HNRNPA2B1	0.387
PTP4A1	0.387
REEP4	0.387
SLC35B3	0.387
PRR15	0.387
RHNO1	0.387
SMIM3	0.387
SLC25A17	0.387
ATP11B	0.387
RAD9A	0.387
FAM107B	0.387

EXOC8	0.387
LPCAT3	0.387
ELP5	0.387
CTNS	0.386
B3GNT9	0.386
TMEM59	0.386
ENTPD2	0.386
C1orf112	0.386
LRRC8A	0.386
CCDC34	0.386
PVT1	0.386
DGKE	0.385
RC3H2	0.385
LIX1L	0.385
B4GALNT1	0.385
ZDHHC20	0.385
SLC30A5	0.385
RABL2A	0.385
SLC48A1	0.385
ITPRIPL1	0.385
ADORA2B	0.385
ADAM17	0.385
MBTD1	0.385
TGFBR3	0.385
DMTF1	0.384
ZNF383	0.384
DENND5B	0.384
CRELD1	0.384
NBPF20	0.384
IFNGR1	0.384
KLF10	0.384
SNAI1	0.384
COQ7	0.383

DHX34	0.383
ELL2	0.383
ZNF581	0.383
MIEF1	0.383
APPL2	0.383
RPAIN	0.383
CLEC2D	0.382
CTCF	0.382
TMEM214	0.382
CYP27A1	0.382
MSMO1	0.382
MTERF1	0.382
REEP6	0.382
MAN1A2	0.382
N4BP2L2	0.381
CLCN6	0.381
STARD10	0.381
NBEAL2	0.381
EXOC3	0.381
RABGGTB	0.381
ABCA7	0.381
DHDDS	0.381
FAM118A	0.381
KAZALD1	0.381
RPL15	0.381
TRAPPC9	0.381
GPR35	0.381
TADA2B	0.381
MTMR3	0.380
PEG3	0.380
TRAPPC8	0.380
IRAK1BP1	0.380
DEGS1	0.379

AKR1C3	0.379
NBPF15	0.379
UBE3A	0.379
PPIA	0.379
UBIAD1	0.379
TDGF1	0.379
TMEM140	0.379
AOC3	0.379
ARFIP2	0.379
IFT140	0.379
NBPF26	0.379
SLC6A9	0.378
SCARF2	0.378
RBM38	0.378
GSTCD	0.378
TSPYL4	0.378
ARMC9	0.378
AZI2	0.378
ALG3	0.378
ZBED3	0.378
RFT1	0.378
SLC30A7	0.377
MANBAL	0.377
RPS23	0.377
LRIG2	0.377
C11orf1	0.377
SPRYD3	0.377
ACVR1B	0.377
SCAP	0.377
STXBP4	0.377
BRD8	0.377
TBC1D16	0.376
ATAD2B	0.376

STARD3	0.376
TIMM17B	0.376
SPRY4	0.376
POGLUT1	0.376
SERPINI1	0.376
RPL10A	0.376
SS18	0.376
TRAPPC10	0.376
SNX29	0.376
PEX19	0.376
FBXL17	0.376
C19orf25	0.376
XYLB	0.376
NDUFA1	0.375
PRNP	0.375
WBP1L	0.375
C5orf63	0.375
VOPP1	0.375
ADCY9	0.375
ZNF556	0.375
EP400	0.375
LHFPL2	0.375
RHPN2	0.375
LYRM2	0.375
GABRE	0.375
ERCC6L2	0.375
RBM41	0.375
NICN1	0.375
WDR83OS	0.375
LEPROT	0.374
ATP6V0B	0.374
NSMAF	0.374
NKD1	0.374

DMXL2	0.374
ZBTB42	0.374
PGBD1	0.374
ROBO1	0.374
REEP2	0.374
PLXNB1	0.374
LNPEP	0.374
SFT2D3	0.374
MED13L	0.374
DIS3L	0.374
MGRN1	0.373
CASD1	0.373
CABLES1	0.373
RPS15A	0.373
PIGH	0.373
KDM2B	0.373
LRRC37A3	0.373
CLDN1	0.373
UBE2M	0.373
MPV17L2	0.372
TLK2	0.372
FNIP2	0.372
WDFY2	0.372
CDS2	0.372
VHL	0.372
YIPF3	0.372
SEMA7A	0.372
RBP1	0.372
MINPP1	0.372
CTDSP2	0.372
PDE7A	0.372
RGP1	0.372
STK24	0.372

RIC1	0.371
TMED7	0.371
TMEM59L	0.371
SIGMAR1	0.371
WDR20	0.371
ZNF276	0.371
E2F6	0.371
NDUFA11	0.370
FAM177A1	0.370
ADAT2	0.370
CERK	0.370
SS18L1	0.370
ETF1	0.370
MTFR1L	0.370
BMF	0.370
TOP3B	0.370
ZXDB	0.370
ERF	0.369
MICALL1	0.369
SHISA5	0.369
KIAA0556	0.369
BCAM	0.369
FANCD2	0.369
DBP	0.369
SLC39A14	0.369
BET1L	0.369
LSR	0.369
ITGA2	0.369
DEF8	0.369
TMEM201	0.369
SLC7A1	0.369
TAF9B	0.369
EEPD1	0.368

MMACHC	0.368
LRRC27	0.368
BTF3	0.368
FAM184A	0.368
RGS3	0.368
SCARB1	0.368
GPAM	0.368
PNRC2	0.368
TRAK2	0.368
MAPK1IP1L	0.368
DLK1	0.368
THRB	0.368
PIP5K1B	0.367
TIMM23	0.367
CRLS1	0.367
PDE4D	0.367
MCEE	0.367
PAGR1	0.367
TBC1D2B	0.367
KXD1	0.367
SUN1	0.367
CNOT6	0.367
KRBA1	0.367
SNAP29	0.366
RASGRF2	0.366
ITGB1BP1	0.366
LCLAT1	0.366
ZNF33B	0.366
CCNC	0.366
CAMK1	0.366
ICE1	0.366
TTC39B	0.366
C16orf70	0.366

PPIP5K1	0.366
CHD3	0.365
HDAC7	0.365
PPP4C	0.365
PGC	0.365
KLHL22	0.365
CES2	0.365
SEC11A	0.365
MLLT10	0.365
AGAP1	0.365
WDR45B	0.365
MBNL3	0.365
MGST2	0.365
CEP57L1	0.364
AIG1	0.364
PLXND1	0.364
RPL23	0.364
CDK19	0.364
EBP	0.364
PGS1	0.364
CDCA7	0.364
PNPO	0.364
EPHX1	0.364
YRDC	0.364
SWSAP1	0.364
GCC1	0.364
RPL35	0.364
ADRA2C	0.364
CASC4	0.364
LRP11	0.364
SLC15A4	0.364
AKAP10	0.364
UHMK1	0.363

CIPC	0.363
KANSL2	0.363
ARHGAP44	0.363
METTL7B	0.363
A1CF	0.363
RAD54L2	0.363
RPS13	0.363
SPIRE2	0.362
PDZK1	0.362
EBPL	0.362
SGPL1	0.362
SOGA1	0.362
GGCX	0.362
SFT2D2	0.362
PTAR1	0.362
NFIX	0.362
TGIF2	0.362
POM121	0.362
ELF1	0.362
SPATA33	0.362
SEMA6A	0.362
TMEM167B	0.362
CREBL2	0.362
PRKAB2	0.362
PLEKHA1	0.362
FBXO45	0.362
ETV4	0.362
SIX5	0.361
EIF1AD	0.361
CDKAL1	0.361
LGR5	0.361
CENPO	0.361
PROSER1	0.361

RALGAPA2	0.361
VSIG10L	0.361
CEP170B	0.361
UNC119	0.361
DUSP3	0.361
DOCK1	0.361
SCAMP1	0.361
DLG3	0.361
CCNY	0.361
C15orf40	0.361
SUFU	0.361
PCSK1N	0.361
